# The global burden of nasopharyngeal carcinoma from 2009 to 2019: an observational study based on the Global Burden of Disease Study 2019

**DOI:** 10.1007/s00405-021-06922-2

**Published:** 2021-06-19

**Authors:** Hao Yu, Xin Yin, Yiran Mao, Meiqin Chen, Qiuying Tang, Senxiang Yan

**Affiliations:** 1 Department of Radiation Oncology, The First Affiliated Hospital, Zhejiang University School of Medicine, #79 Qingchun Road, Hangzhou, 310003 Zhejiang People’s Republic of China; 2 School of Life Sciences, Tsinghua University, 30 Shuangqing Road, Haidian District, Beijing, 100084 People’s Republic of China; 3 Department of Radiation Oncology, Affiliated Jinhua Hospital, College of Medicine, Zhejiang University, Jinhua, 321000 Zhejiang People’s Republic of China

**Keywords:** Nasopharyngeal carcinoma (NPC), Global Burden of Disease (GBD), Incidence, Mortality, Age-standardized rate (ASR), Estimated annual percentage change (EAPC)

## Abstract

**Purpose:**

The incidence and mortality rate of nasopharyngeal carcinoma (NPC) has changed in recent years. Our goal is to determine the epidemiological pattern of NPC to help policymakers allocate limited medical resources.

**Methods:**

Detailed information about NPC from 2009 to 2019 was collected from the Global Burden of Disease 2019 database. Age-standardized rates (ASRs) and corresponding estimated annual percentage changes (EAPCs) were calculated to assess NPC’s incidence and mortality trends.

**Results:**

Globally, there was a consistent increase in the NPC incidence cases from 2009 to 2019 (from 121.65 × 10^3^ cases in 2009 to 176.50 × 10^3^ cases in 2019, increasing by 45.09%). The age-standardized incidence rate (ASIR) of NPC increased from 1.81 in 2009 to 2.12 in 2019 (EAPC = 1.59, 95% CI 1.36–1.81). On the contrary, the mortality of NPC showed a downward trend (ASDR: 0.93 in 2009 and 0.86 in 2019; EAPC = − 0.63, 95% CI − 0.78 to − 0.48), and it was negatively correlated with the social demographic index (SDI) in most regions. Both incidence and mortality rates of high-incidence territories tended to be stable or decline. Males had significantly higher incidence and mortality of NPC than females. The number of patients with onset age greater than 50 years old accounted for the highest proportion. We found that smoking, occupational exposure to formaldehyde, and alcohol use were the main risk factors for NPC-related mortality.

**Conclusion:**

Globally, the incidence rate of NPC has been slightly increasing, while the mortality and disability-adjusted life years (DALYs) have been decreasing. NPC burden in high-middle and middle SDI areas was the heaviest. The current prevention strategy should be repositioned, and some countries should formulate more targeted approaches to reduce the current burden of NPC.

**Supplementary Information:**

The online version contains supplementary material available at 10.1007/s00405-021-06922-2.

## Introduction

Nasopharyngeal carcinoma (NPC) originates from the nasopharyngeal epithelium and is divided into three major pathological subtypes: keratinizing squamous, non-keratinizing squamous and basaloid squamous cell carcinoma. Among them, non-keratinizing NPC can be divided into differentiated and undifferentiated tumors [1]. In the high incidence area of NPC, non-keratinizing carcinoma accounts for the majority, while in the low incidence area, keratinizing carcinoma is the primary type [2, 3]. Compared with other cancers, NPC has a unique distribution pattern. In 2018, 129,000 new cases and 73,000 deaths of NPC were reported worldwide [4]. The geographic distribution of these cases was extremely unbalanced, with more than 70% of new cases occurring in East and Southeast Asia, while in most other regions, the age-standardized incidence rate of NPC in both males and females was less than 1/100,000 person-years [1]. The incidence rate of NPC in males is 2–3 times higher than females in almost all the populations surveyed [5].

The etiology of NPC is complex, including Epstein–Barr virus (EBV) infection, host genetics, and environmental factors [5]. EBV is ubiquitous in NPC cells, most closely related to the undifferentiated histological type of NPC, which is most common in southern China and Southeast Asia [6, 7]. However, the carcinogenic mechanism of EBV remains unclear, possibly due to the abnormal interaction between latent viral infection in epithelial cells and existing precancerous genetic changes [8]. In addition to viral infection, host genetic factors are also crucial causal agents for NPC. The human leukocyte antigen gene (HLA) located in the MHC region on chromosome 6p21 has been widely recognized as a major risk site for NPC [9]. The main environmental factors for NPC include smoking [10], alcohol consumption [11], eating salted fish and salted products [12, 13], while eating fresh fruits and vegetables is a protective factor for NPC [14].

To accommodate the rapid change in the epidemiological characteristics of NPC, a complete study to examine the epidemiological patterns of NPC at multiple levels is required, to comprehensively assess the distribution and development trend of NPC in different countries. In this study, we detailed NPC statistics of 204 countries or territories worldwide from 2009 to 2019, aiming to help policymakers assess the burden of disease and allocate the limited resources of the medical system accordingly.

## Materials and methods

### Data sources

The Global Burden of Disease (GBD) database contains statistics on 369 diseases in 204 countries or territories [15]. The data including incidence numbers, deaths, disability-adjusted life years (DALYs), and corresponding age-standardized rates (ASRs) were downloaded from the Global Health Data Exchange (GHDx) query tool (http://ghdx.healthdata.org/gbd-results-tool). Information on gender, age, and potential risk factors associated with NPC was also downloaded to assess their impact on disease burden. Social demographic index (SDI) was used to divide countries or territories into five categories (high SDI, high-middle SDI, middle SDI, low-middle SDI and low SDI region). SDI values range from 0 to 1, which indicates a country’s degree of development based on the level of per capita income, education and total fertility rate [15].

### Statistical analysis

Annual incidence cases, deaths, DALYs, ASRs and corresponding estimated annual percentage changes (EAPCs) were used to assess trends in the incidence and mortality rate of NPC. DALYs are composed of years of life lost (YLLs) and years lived with disability (YLDs), and the corresponding calculation formula is DALYs = YLLs + YLDs. One DALY can be regarded as 1 lost year of "healthy life" [15]. ASRs include age-standardized incidence rate (ASIR), age-standardized death rate (ASDR) and age-standardized DALY rate. The ASR provided in GHDx is considered an objective indicator to quantify trends in cancer incidence. Standardization is required for comparison among several differently age-structured populations or for a certain population over time with its time-dependent age profiles. The trend of ASR can be reflected by EAPC value, which is often used to measure trends in disease and mortality rates, using a linear model on the log of the ASR [16]. According to the following regression model: $y={\beta }_{0}+{\beta }_{1}x+\varepsilon$, where $y$ represents ln(ASR) and $x$ refers to the calendar year, $\mathrm{EAPC}=100\times (\mathrm{exp}\left({\beta }_{1}\right)-1)$ and its 95% confidence interval (CI) can be obtained. If the EAPC value and its lower limit of 95% CI are both positive, ASR is considered rising. Conversely, if the EAPC value and its upper limit of 95% CI are both negative, ASR shows a downward trend. Otherwise, ASR is considered stable. Finally, to examine the correlation between the trend of ASRs and the degree of social development, we calculated the Pearson correlation coefficient between EAPCs and SDI values.

### Data visualization

All data analysis was based on open-source software R (version 3.6.3). Data cleaning was conducted using packages including dplyr and tidyr. Data visualization was performed using packages including ggplot2, Rcolor Brewer, and cowplot. Stacked histograms were used to show the trends of NPC incidence, death and DALY from 2009 to 2019. The world map was used to display the NPC burden of 204 countries or territories visually. Bubble plots and scatter plots were used and regression curves were added to analyze the correlation between ASRs and SDI values as well as that between EAPCs and SDI values. Histograms were used to show the differences in incidence and mortality between males and females in territories with high incidence of NPC. The dynamic distribution of age composition and mortality-related risk factors in NPC patients was represented by area graphs.

## Results

### Change in the incidence of NPC

**Fig. 1:**
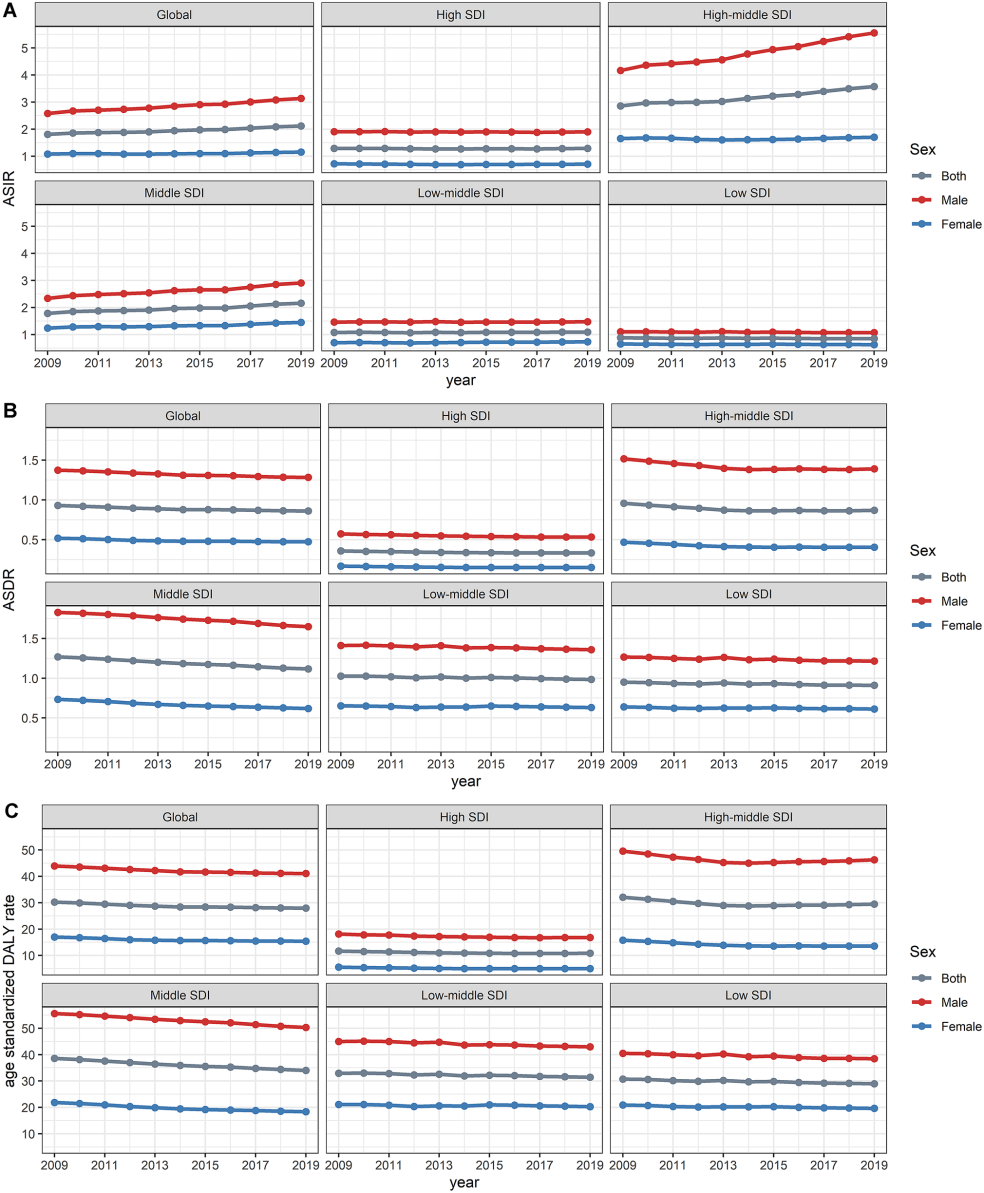
The change trends of NPC’s ASIR, ASDR, and age-standardized DALY rate from 2009 to 2019. **A** The change trends of ASIR. **B** The change trends of ASDR. **C** The change trends of age-standardized DALY rate

**Fig. 2:**
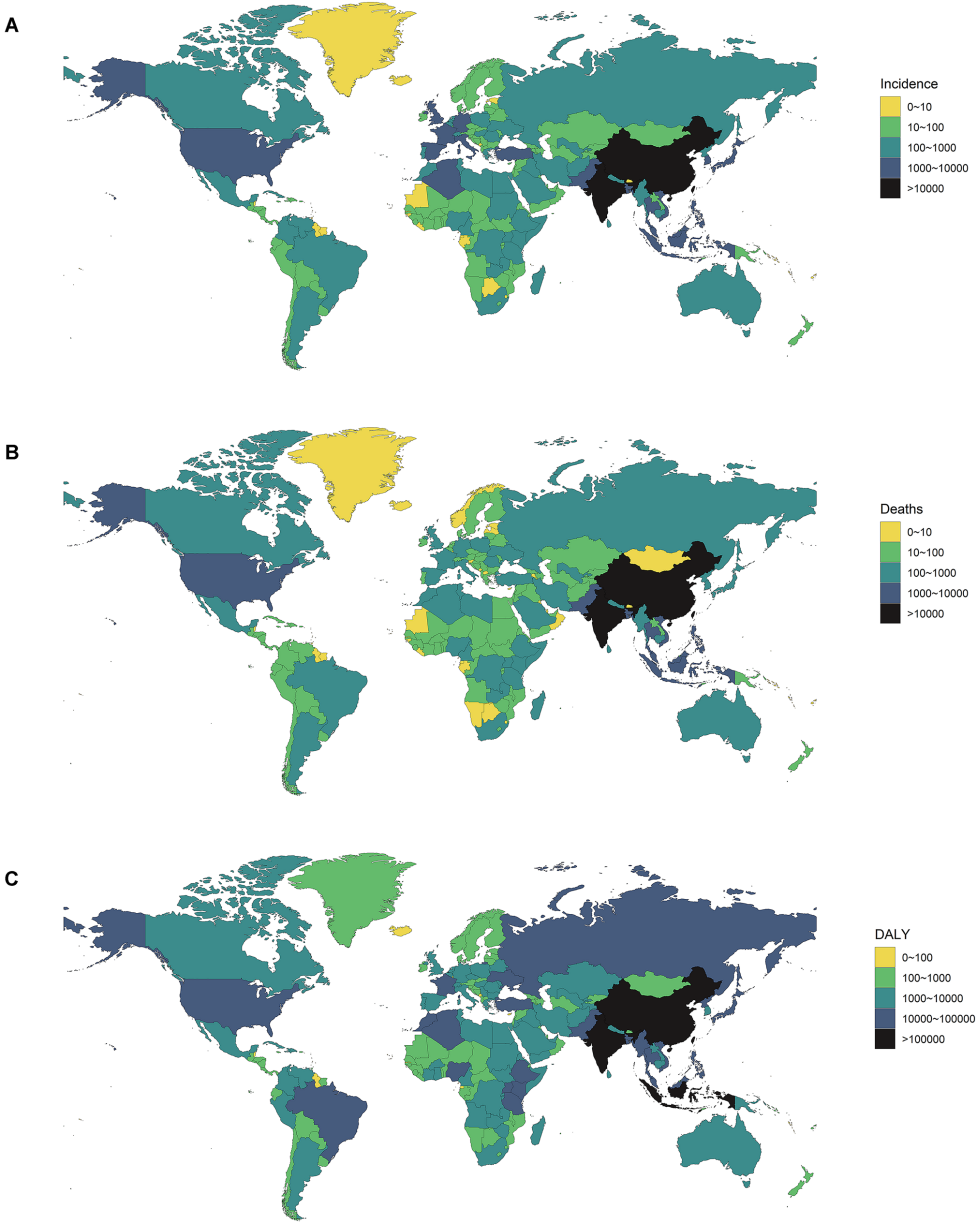
The global disease burden of NPC for both sexes in 204 countries or territories. **A** The incidence cases of NPC in 2019. **B** The deaths of NPC in 2019. **C** The DALYs of NPC in 2019

**Fig. 3:**
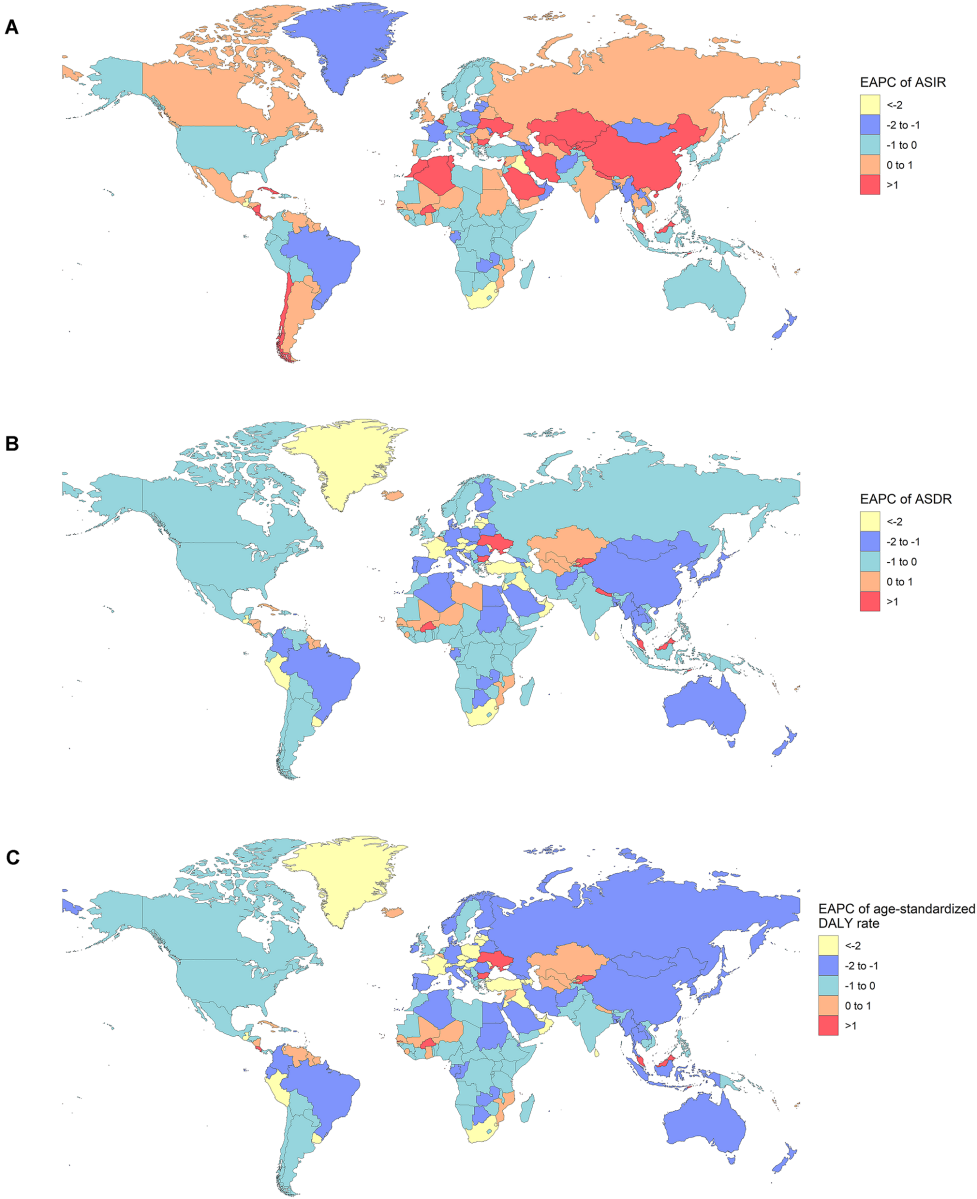
The EAPCs of NPC in 204 countries or territories. **A** The EAPC of ASIR from 2009 to 2019. **B** The EAPC of ASDR from 2009 to 2019. **C** The EAPC of age-standardized DALY rate from 2009 to 2019

There was a consistent yearly increase in the NPC incidence from 2009 to 2019 worldwide (from 121.65 × 10^3^ cases in 2009 to 176.50 × 10^3^ cases in 2019, increasing by 45.09%) (Fig. 1A) (Table 1). There were more male patients than female ones (male to female ratio = 2.27:1 in 2009, and 2.59:1 in 2019). The global incidence rate of NPC also showed an increasing trend (ASIR: 1.81 in 2009 and 2.12 in 2019, EAPC = 1.59, 95% CI 1.36–1.81). Subgroup analysis by SDI demonstrated that high-middle SDI area had the highest burden of NPC (incidence cases: 46.12 × 10^3^ in 2009 and 68.02 × 10^3^ in 2019; ASIR: 2.86 in 2009 and 3.58 in 2019, EAPC = 2.46, 95% CI 2.13–2.79). ASIR decreased the fastest in the low SDI areas (ASIR: 0.88 in 2009 and 0.85 in 2019, EAPC = − 0.28, 95% CI − 0.47 to − 0.08), and plateaued in high SDI areas (ASIR: 1.29 in 2009 and 1.29 in 2019, EAPC = 0.00, 95% CI − 0.17 to 0.17). At the intercontinental level, East Asia and South Asia had the most incidence cases (East Asia: 71.34 × 10^3^ cases in 2009 and 113.40 × 10^3^ cases in 2019; South Asia: 11.92 × 10^3^ cases in 2009 and 15.78 × 10^3^ cases in 2019). ASIR had increased in East Asia, Central Asia, the Middle East, and Eastern Europe, and the two fastest-growing areas were East Asia and Eastern Europe. ASIR tended to be stable or decreased in most other regions, especially in southern Sub-Sahara Africa and Tropical Latin America (EAPC = − 1.58 and − 1.09, respectively) (Fig. 3A). At the national or regional level, the top three countries of incidence cases were China, India and Japan, all located in Asia (68.78 × 10^3^, 9.13 × 10^3^, and 3.74 × 10^3^ cases in 2009, respectively; 110.43 × 10^3^, 12.21 × 10^3^, 3.98 × 10^3^ cases in 2019, respectively) (Fig. 2A) (Tables S1, S7). Singapore and Taiwan (province of China) had the highest ASIR both in 2009 and 2019 (Singapore: ASIR = 12.64 in 2009 and 10.81 in 2019; Taiwan: ASIR = 6.77 in 2009 and 7.14 in 2019) (Tables S4, S10, Fig. S1A). Ukraine had the most significant increase in ASIR (EAPC = 3.68, 95% CI 2.49–4.87) (Table S13).

**Table 1:** The incident cases and ASIR of NPC in 2009/2019 and its temporal trends

	2009		2019		2009–2019
	Incidence cases No *10^3^ (95% CI)	ASIR/100,000 No. (95% CI)	Incidence cases No *10^3^ (95% CI)	ASIR/100,000 No. (95% CI)	EAPC No. (95% CI)
Global	121.65 (114.34–130.12)	1.81 (1.70–1.93)	176.50 (156.05–199.92)	2.12 (1.87–2.40)	1.59 (1.36–1.81)
Male	84.48 (78.14–91.91)	2.58 (2.40–2.80)	127.28 (108.04–148.52)	3.14 (2.67–3.65)	1.90 (1.73–2.08)
Female	37.17 (34.28–40.10)	1.08 (1.00–1.16)	49.22 (42.60–56.98)	1.16 (1.00–1.34)	0.78 (0.40–1.17)
High SDI	16.76 (16.15–17.33)	1.29 (1.25–1.34)	19.27 (17.28–21.48)	1.29 (1.16–1.44)	0.00 (− 0.17 to 0.17)
High-middle SDI	46.12 (41.54–51.30)	2.86 (2.58–3.17)	68.02 (56.47–81.57)	3.58 (2.98–4.28)	2.46 (2.13–2.79)
Middle SDI	37.03 (34.41–40.34)	1.78 (1.66–1.93)	57.53 (50.14–65.91)	2.16 (1.89–2.47)	1.80 (1.41–2.18)
Low-middle SDI	12.43 (11.43–13.53)	1.08 (0.99–1.17)	16.59 (15.07–18.48)	1.09 (1.00–1.22)	0.21 (0.07–0.35)
Low SDI	4.10 (3.64–4.58)	0.88 (0.78–0.98)	5.38 (4.77–5.97)	0.85 (0.75–0.94)	− 0.28 (− 0.47 to − 0.08)
Andean Latin America	0.08 (0.07–0.09)	0.18 (0.16–0.20)	0.10 (0.08–0.13)	0.18 (0.14–0.22)	− 0.55 (− 0.76 to − 0.35)
Australasia	0.28 (0.26–0.30)	0.83 (0.78–0.90)	0.30 (0.24–0.38)	0.75 (0.60–0.93)	− 0.84 (− 1.27 to − 0.41)
Caribbean	0.26 (0.24–0.29)	0.62 (0.56–0.68)	0.33 (0.28–0.39)	0.65 (0.55–0.75)	0.62 (0.42–0.82)
Central Asia	0.29 (0.28–0.30)	0.42 (0.41–0.44)	0.39 (0.35–0.45)	0.46 (0.41–0.52)	0.99 (0.43–1.55)
Central Europe	1.04 (1.00–1.08)	0.64 (0.62–0.66)	1.03 (0.90–1.19)	0.62 (0.54–0.72)	− 0.29 (− 0.53 to − 0.04)
Central Latin America	0.43 (0.41–0.44)	0.23 (0.22–0.24)	0.56 (0.48–0.67)	0.23 (0.20–0.27)	− 0.01 (− 0.41 to 0.40)
Central Sub-Saharan Africa	0.24 (0.19–0.29)	0.52 (0.42–0.64)	0.33 (0.25–0.42)	0.51 (0.39–0.65)	− 0.40 (− 0.51 to − 0.30)
East Asia	71.34 (64.54–79.06)	4.21 (3.82–4.64)	113.40 (93.19–135.38)	5.61 (4.66–6.68)	2.74 (2.44–3.04)
Eastern Europe	1.35 (1.30–1.41)	0.47 (0.46–0.49)	1.51 (1.33–1.70)	0.53 (0.47–0.59)	1.84 (0.82–2.86)
Eastern Sub-Saharan Africa	1.89 (1.49–2.21)	1.25 (0.98–1.47)	2.54 (1.91–3.11)	1.21 (0.90–1.47)	− 0.36 (− 0.44 to − 0.28)
High-income Asia Pacific	5.29 (4.95–5.59)	1.80 (1.70–1.89)	5.98 (5.14–6.93)	1.73 (1.50–1.98)	− 0.63 (− 0.84 to − 0.42)
High-income North America	3.13 (3.01–3.24)	0.71 (0.68–0.73)	3.53 (3.03–4.10)	0.69 (0.60–0.80)	− 0.13 (− 0.26 to 0.00)
North Africa and Middle East	4.44 (3.94–4.99)	1.14 (1.01–1.27)	6.64 (5.78–7.69)	1.25 (1.09–1.44)	0.90 (0.79–1.00)
Oceania	0.08 (0.06–0.10)	1.26 (0.95–1.59)	0.11 (0.08–0.14)	1.28 (0.95–1.64)	0.04 (− 0.11 to 0.20)
South Asia	11.92 (10.84–13.13)	1.02 (0.93–1.12)	15.78 (13.77–18.20)	1.01 (0.88–1.16)	− 0.10 (− 0.20 to 0.00)
Southeast Asia	9.85 (9.04–10.73)	1.93 (1.77–2.09)	13.12 (11.40–15.08)	1.95 (1.70–2.23)	0.16 (− 0.05 to 0.36)
Southern Latin America	0.18 (0.17–0.20)	0.28 (0.26–0.30)	0.23 (0.18–0.29)	0.29 (0.23–0.37)	0.41 (− 0.10 to 0.93)
Southern Sub-Saharan Africa	0.28 (0.26–0.31)	0.59 (0.55–0.64)	0.31 (0.27–0.34)	0.50 (0.45–0.55)	− 1.58 (− 1.85 to − 1.31)
Tropical Latin America	0.59 (0.57–0.61)	0.30 (0.29–0.31)	0.69 (0.64–0.73)	0.28 (0.26–0.30)	− 1.09 (− 1.30 to − 0.87)
Western Europe	7.67 (7.32–8.02)	1.26 (1.20–1.32)	8.24 (7.01–9.63)	1.21 (1.03–1.43)	− 0.28 (− 0.52 to − 0.05)
Western Sub-Saharan Africa	1.01 (0.80–1.29)	0.59 (0.47–0.74)	1.38 (1.09–1.71)	0.57 (0.46–0.71)	− 0.28 (− 0.67 to 0.11)

*ASIR* age-standardized incidence rate

### Change in NPC mortality

Globally, although the NPC-related deaths were increasing yearly, the ASDR was declining (ASDR: 0.93 in 2009 and 0.86 in 2019; EAPC = − 0.63, 95% CI − 0.78 to − 0.48) (Fig. 1B) (Table 2). The number of deaths in males was higher than that in females (male to female ratio = 2.46:1 in 2009, and 2.51:1 in 2019). Subgroup analysis by SDI demonstrated that the middle SDI region had the most NPC-related deaths (24.62 × 10^3^ cases in 2009 and 28.75 × 10^3^ cases in 2019). ASDR decreased in all five SDI regions, especially in middle SDI and high SDI areas (EAPC = − 1.28 and − 0.54, respectively). At the intercontinental level, three regions with the highest number of NPC-related deaths were all located in Asia (deaths in 2019: East Asia: 30.10 × 10^3^; South Asia: 14.66 × 10^3^; Southeast Asia: 11.26 × 10^3^). ASDR in most areas showed a downward trend, among which Tropical Latin America and southern Sub-Sahara Africa showed the fastest decline (Tropical Latin America: EAPC = − 1.74, 95% CI − 1.92 to − 1.56; Southern Sub-Saharan Africa: EAPC = − 1.70, 95% CI − 2.00 to − 1.40). At the national or regional level, China, India and Indonesia had the largest number of deaths (26.03 × 10^3^, 8.60 × 10^3^, and 2.69 × 10^3^ cases in 2009, respectively; 28.66 × 10^3^, 11.36 × 10^3^, 3.22 × 10^3^ cases in 2019, respectively) (Fig. 2B) (Tables S2, S8). Greenland and Malaysia were two regions with the highest ASDR both in 2009 and 2019 (Greenland: ASDR = 5.69 in 2009 and 4.68 in 2019; Malaysia: ASDR = 4.21 in 2009 and 4.76 in 2019) (Tables S5, S11, Fig. S1B). Although the EAPC values of a small part of regions were positive, the lower limits of 95% CI of EAPC values of all countries or territories were negative. Therefore, ASDR of all countries and territories in the world tended to be stable or declining. ASDR of high incidence territories of NPC such as East Asia, Southeast Asia, South Asia, North Africa and the Middle East also decreased to varying degrees (Fig. 3B) (Table S14).

**Table 2:** The deaths and ASDR of NPC in 2009/2019 and its temporal trends

	2009		2019		2009–2019
	Deaths cases No *10^3^ (95% CI)	ASDR/100,000 No. (95% CI)	Deaths cases No *10^3^ (95% CI)	ASDR/100,000 No. (95% CI)	EAPC No. (95% CI)
Global	60.78 (57.52–64.14)	0.93 (0.88–0.98)	71.61 (65.44–77.62)	0.86 (0.79–0.93)	− 0.63 (− 0.78 to − 0.48)
Male	43.21 (40.60–46.14)	1.38 (1.29–1.47)	51.22 (45.97–56.95)	1.28 (1.15–1.43)	− 0.64 (− 0.76 to − 0.53)
Female	17.57 (16.09–18.84)	0.52 (0.48–0.56)	20.39 (18.16–22.78)	0.47 (0.42–0.53)	− 0.61 (− 0.84 to − 0.37)
High SDI	4.98 (4.78–5.11)	0.36 (0.35–0.37)	5.55 (5.14–5.98)	0.33 (0.31–0.36)	− 0.54 (− 0.81 to − 0.27)
High-middle SDI	15.42 (14.34–16.71)	0.96 (0.89–1.04)	17.22 (14.92–19.68)	0.87 (0.75–0.99)	− 0.53 (− 0.98 to − 0.07)
Middle SDI	24.62 (23.10–26.53)	1.27 (1.19–1.36)	28.75 (25.63–32.05)	1.12 (1.00–1.24)	− 1.28 (− 1.35 to − 1.20)
Low-middle SDI	11.47 (10.54–12.45)	1.03 (0.94–1.11)	14.51 (13.15–16.11)	0.98 (0.89–1.09)	− 0.37 (− 0.54 to − 0.19)
Low SDI	4.25 (3.82–4.73)	0.95 (0.86–1.06)	5.54 (4.90–6.23)	0.91 (0.81–1.02)	− 0.35 (− 0.53 to − 0.17)
Andean Latin America	0.07 (0.06–0.08)	0.18 (0.16–0.19)	0.09 (0.07–0.11)	0.16 (0.13–0.19)	− 1.20 (− 1.35 to − 1.04)
Australasia	0.12 (0.11–0.12)	0.33 (0.31–0.35)	0.13 (0.12–0.15)	0.30 (0.27–0.33)	− 0.94 (− 1.18 to − 0.70)
Caribbean	0.22 (0.20–0.24)	0.52 (0.47–0.58)	0.27 (0.23–0.31)	0.52 (0.44–0.60)	0.18 (− 0.08 to 0.43)
Central Asia	0.26 (0.25–0.27)	0.40 (0.38–0.41)	0.34 (0.30–0.38)	0.41 (0.36–0.46)	0.53 (− 0.06 to 1.13)
Central Europe	0.80 (0.77–0.82)	0.46 (0.45–0.48)	0.74 (0.64–0.84)	0.40 (0.35–0.45)	− 1.52 (− 1.77 to − 1.28)
Central Latin America	0.36 (0.34–0.37)	0.20 (0.19–0.21)	0.45 (0.38–0.53)	0.19 (0.16–0.22)	− 0.79 (− 1.24 to − 0.35)
Central Sub-Saharan Africa	0.23 (0.19–0.28)	0.53 (0.43–0.65)	0.32 (0.24–0.40)	0.51 (0.39–0.66)	− 0.43 (− 0.52 to − 0.33)
East Asia	27.34 (25.11–29.88)	1.71 (1.57–1.86)	30.10 (25.23–35.36)	1.45 (1.22–1.70)	− 1.68 (− 1.92 to − 1.44)
Eastern Europe	0.95 (0.93–0.97)	0.32 (0.31–0.33)	0.96 (0.84–1.09)	0.31 (0.27–0.35)	0.07 (− 0.66–0.81)
Eastern Sub-Saharan Africa	1.80 (1.41–2.10)	1.25 (0.97–1.46)	2.41 (1.81–2.94)	1.20 (0.91–1.46)	− 0.40 (− 0.45 to − 0.35)
High-income Asia Pacific	1.16 (1.08–1.21)	0.36 (0.34–0.37)	1.30 (1.17–1.41)	0.32 (0.29–0.35)	− 1.31 (− 1.52 to − 1.10)
High-income North America	1.09 (1.05–1.12)	0.23 (0.23–0.24)	1.27 (1.21–1.32)	0.22 (0.21–0.23)	− 0.34 (− 0.54 to − 0.14)
North Africa and Middle East	2.40 (2.11–2.72)	0.69 (0.61–0.78)	2.95 (2.57–3.37)	0.61 (0.54–0.70)	− 1.10 (− 1.18 to − 1.02)
Oceania	0.07 (0.05–0.09)	1.23 (0.93–1.55)	0.10 (0.08–0.13)	1.25 (0.94–1.60)	0.06 (− 0.10–0.22)
South Asia	11.22 (10.24–12.33)	0.99 (0.90–1.08)	14.66 (12.96–16.86)	0.96 (0.85–1.10)	− 0.28 (− 0.39 to − 0.17)
Southeast Asia	8.90 (8.18–9.70)	1.83 (1.68–1.98)	11.26 (9.87–12.85)	1.74 (1.53–1.98)	− 0.43 (− 0.58 to − 0.28)
Southern Latin America	0.14 (0.13–0.14)	0.21 (0.19–0.22)	0.15 (0.14–0.16)	0.19 (0.17–0.20)	− 0.97 (− 1.43 to − 0.51)
Southern Sub-Saharan Africa	0.27 (0.25–0.29)	0.59 (0.55–0.63)	0.29 (0.26–0.32)	0.49 (0.44–0.54)	− 1.70 (− 2.00 to − 1.40)
Tropical Latin America	0.48 (0.47–0.50)	0.25 (0.25–0.26)	0.54 (0.50–0.57)	0.22 (0.20–0.23)	− 1.74 (− 1.92 to − 1.56)
Western Europe	1.95 (1.88–2.01)	0.29 (0.28–0.30)	1.99 (1.87–2.11)	0.26 (0.24–0.27)	− 1.19 (− 1.42 to − 0.96)
Western Sub-Saharan Africa	0.96 (0.75–1.22)	0.58 (0.46–0.73)	1.30 (1.01–1.59)	0.57 (0.45–0.69)	− 0.33 (− 0.71 to 0.05)

*ASDR* age-standardized death rate

### Change in DALYs for NPC

Globally, the DALYs were increasing from 2046.98 × 10^3^ in 2009 to 2335.10 × 10^3^ in 2019, while the age-standardized DALY rate declined (30.22 in 2009 and 27.98 in 2019, EAPC = − 0.60, 95% CI − 0.79 to − 0.42) (Fig. 1C, Table 3). Males were still the main component (male to female ratio = 2.50:1 in 2009 and 2.59:1 in 2019). Subgroup analysis by SDI demonstrated that the middle SDI region had the highest DALY (814.13 × 10^3^ in 2009 and 914.42 × 10^3^ in 2019). The age-standardized DALY rate decreased in all regions, especially in middle SDI and high SDI areas (EAPC = − 1.21 and − 0.56, respectively). At the intercontinental level, DALYs were all gradually increasing. East Asia had the highest DALY (902.48 × 10^3^ in 2009 and 958.33 × 10^3^ in 2019). However, the age-standardized DALY rate decreased in most regions including East Asia, especially in southern Sub-Saharan Africa (EAPC = − 1.83, 95% CI − 2.03 to − 1.63). At the national or regional level, China, India and Indonesia had the most DALY (858.85 × 10^3^, 306.54 × 10^3^, and 88.62 × 10^3^ in 2009, respectively; 912.11 × 10^3^, 388.09 × 10^3^, 100.80 × 10^3^ in 2019, respectively) (Fig. 2C) (Tables S3, S9). Malaysia and Greenland were two countries with the highest age-standardized DALY rates both in 2009 and 2019 (Greenland: 171.99 in 2009 and 137.74 in 2019; Malaysia: 131.31 in 2009 and 152.29 in 2019) (Tables S6, S12, Fig. S1C). Just like ASDR, the Age-standardized DALY rate plateaued or decreased in all countries or territories worldwide (Fig. 3C) (Table S15).

**Table 3:** The DALYs and age-standardized DALY rate of NPC in 2009/2019 and its temporal trends

	2009		2019		2009–2019
	DALY No *10^3^ (95% CI)	Age-standardized DALY rate/100,000 No. (95% CI)	DALY No *10^3^ (95% CI)	Age-standardized DALY rate/100,000 No. (95% CI)	EAPC No. (95% CI)
Global	2046.98 (1935.69–2172.48)	30.22 (28.59–32.04)	2335.10 (2139.75–2536.66)	27.98 (25.65–30.37)	− 0.60 (− 0.79 to − 0.42)
Male	1461.53 (1371.90–1569.63)	43.94 (41.29–47.08)	1684.24 (1514.43–1863.91)	41.09 (36.98–45.44)	− 0.57 (− 0.72 to − 0.41)
Female	585.45 (535.67–629.28)	17.01 (15.57–18.30)	650.86 (580.29–726.36)	15.39 (13.73–17.16)	− 0.68 (− 0.94 to − 0.41)
High SDI	150.17 (145.09–155.05)	11.67 (11.30–12.04)	159.39 (146.90–173.32)	10.82 (9.95–11.78)	− 0.56 (− 0.86 to − 0.26)
High-middle SDI	519.63 (484.58–562.70)	32.08 (29.94–34.74)	563.90 (492.01–643.06)	29.49 (25.76–33.62)	− 0.27 (− 0.79 to 0.26)
Middle SDI	814.13 (761.24–884.84)	38.58 (36.17–41.73)	914.42 (819.01–1020.65)	34.02 (30.52–37.87)	− 1.21 (− 1.30 to − 1.12)
Low-middle SDI	404.27 (369.74–441.68)	32.96 (30.20–35.93)	494.50 (446.24–550.74)	31.42 (28.40–34.96)	− 0.47 (− 0.63 to − 0.31)
Low SDI	157.82 (140.93–176.69)	30.74 (27.58–34.20)	201.75 (177.99–227.16)	28.97 (25.53–32.56)	− 0.52 (− 0.70 to − 0.35)
Andean Latin America	2.21 (1.97–2.44)	4.93 (4.38–5.47)	2.55 (2.03–3.16)	4.30 (3.42–5.33)	− 1.52 (− 1.77 to − 1.26)
Australasia	3.47 (3.26–3.68)	10.45 (9.83–11.07)	3.73 (3.36–4.13)	9.37 (8.43–10.35)	− 0.98 (− 1.23 to − 0.73)
Caribbean	6.44 (5.68–7.25)	15.04 (13.29–16.94)	7.76 (6.53–9.12)	15.16 (12.72–17.85)	0.30 (0.03–0.57)
Central Asia	9.57 (9.18–9.99)	13.15 (12.64–13.71)	12.21 (10.78–13.87)	13.61 (12.03–15.39)	0.44 (− 0.13 to 1.01)
Central Europe	24.58 (23.82–25.38)	15.10 (14.66–15.58)	21.53 (18.62–24.72)	12.83 (11.11–14.77)	− 1.64 (− 1.94 to − 1.33)
Central Latin America	11.07 (10.70–11.39)	5.80 (5.60–5.98)	13.19 (11.10–15.76)	5.33 (4.50–6.35)	− 0.73 (− 1.13 to − 0.33)
Central Sub-Saharan Africa	8.22 (6.60–10.05)	15.85 (12.78–19.43)	11.21 (8.56–14.14)	15.22 (11.66–19.44)	− 0.48 (− 0.58 to − 0.37)
East Asia	902.48 (831.26–988.48)	53.20 (49.08–58.20)	958.33 (810.44–1124.17)	46.29 (39.30–54.04)	− 1.40 (− 1.76 to − 1.04)
Eastern Europe	30.88 (30.17–31.68)	10.84 (10.60–11.11)	30.89 (27.09–35.03)	10.68 (9.38–12.08)	0.39 (− 0.45 to 1.23)
Eastern Sub-Saharan Africa	67.97 (53.54–79.34)	40.48 (31.60–47.32)	90.08 (67.60–110.87)	38.43 (28.90–46.98)	− 0.50 (− 0.56 to − 0.44)
High-income Asia Pacific	31.32 (29.82–32.72)	10.89 (10.43–11.35)	31.88 (29.13–34.73)	9.64 (8.84–10.53)	− 1.47 (− 1.69 to − 1.26)
High-income North America	33.26 (32.39–34.22)	7.49 (7.29–7.70)	36.56 (34.98–38.17)	7.07 (6.77–7.37)	− 0.41 (− 0.62 to − 0.21)
North Africa and Middle East	85.93 (74.95–97.76)	21.79 (19.05–24.72)	102.67 (89.16–118.58)	19.13 (16.69–21.98)	− 1.26 (− 1.36 to − 1.15)
Oceania	2.55 (1.85–3.36)	37.87 (28.04–48.97)	3.57 (2.56–4.74)	38.25 (27.89–49.97)	− 0.01 (− 0.17 to 0.16)
South Asia	404.50 (368.60–445.60)	32.24 (29.45–35.47)	508.46 (449.58–584.00)	31.11 (27.48–35.71)	− 0.40 (− 0.50 to − 0.29)
Southeast Asia	299.55 (273.30–327.78)	55.30 (50.66–60.38)	364.17 (316.98–418.04)	52.27 (45.66–59.98)	− 0.49 (− 0.67 to − 0.32)
Southern Latin America	3.99 (3.77–4.22)	6.17 (5.83–6.52)	4.27 (3.91–4.66)	5.54 (5.08–6.05)	− 1.05 (− 1.53 to − 0.57)
Southern Sub-Saharan Africa	8.89 (8.22–9.56)	17.17 (15.98–18.41)	9.27 (8.26–10.36)	14.16 (12.66–15.76)	− 1.83 (− 2.03 to − 1.63)
Tropical Latin America	17.55 (16.98–18.15)	8.77 (8.48–9.06)	18.58 (17.42–19.70)	7.47 (7.00–7.92)	− 1.76 (− 1.97 to − 1.55)
Western Europe	57.43 (55.22–59.59)	9.50 (9.16–9.85)	56.03 (52.22–59.85)	8.36 (7.80–8.93)	− 1.27 (− 1.58 to − 0.96)
Western Sub-Saharan Africa	35.12 (27.27–45.15)	18.42 (14.37–23.46)	48.18 (36.97–59.61)	18.04 (13.94–22.09)	− 0.26 (− 0.68 to 0.17)

*DALY* Disability-adjusted life year

### The correlation between SDI and global NPC burden

**Fig. 4:**
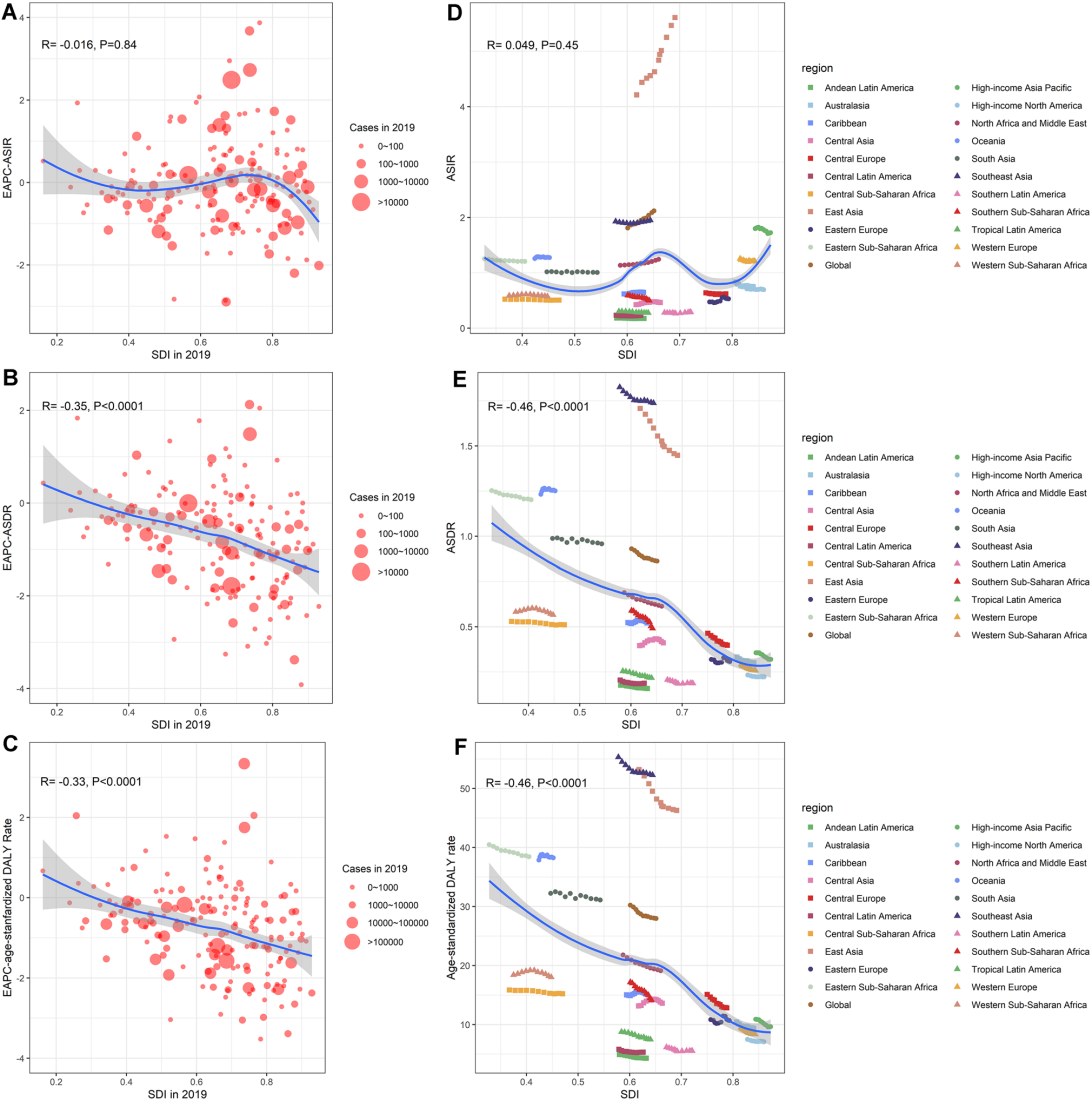
Correlation between the global burden of NPC and SDI. **A**–**C** The correlation between EAPC of ASRs and SDI of 2019 in 204 countries or territories. **D**–**F** The change trends and correlation of ASRs and SDI from 2009 to 2019 in 21 regions

We explored the correlation between SDI in 2019 and EAPCs of ASRs in 204 countries or territories. The results demonstrated that negative correlation occurred between SDI and EAPC values of all ASRs, and the correlation between SDI and EAPCs of ASDR/age-standardized DALY rate was statistically significant (correlation coefficient = − 0.35 and − 0.33, respectively. All *P* values < 0.0001) (Fig. 4A–C). In Fig. 4A, there was a turning point when SDI value was between 0.6 and 0.8, which may be associated with the higher value of EAPC of ASIR in high-middle SDI region (EAPC = 2.46, 95% CI 2.13–2.79). We further explored the relationship between SDI in 2019 and NPC burden in 21 geographical regions from 2009 to 2019 (Fig. 4D–F). In general, there was a statistically significant negative linear relationship between SDI and ASDR/age-standardized DALY rate (correlation coefficient = − 0.46 and − 0.46, respectively. All *P* values < 0.0001). However, the relationship between SDI and ASIR was positive (correlation coefficient = 0.049, *P* value > 0.1). There was a peak in the curve when SDI value was between 0.6 and 0.7, which may be related to the data of East Asia.

### NPC burden of high-incidence territories

**Fig. 5:**
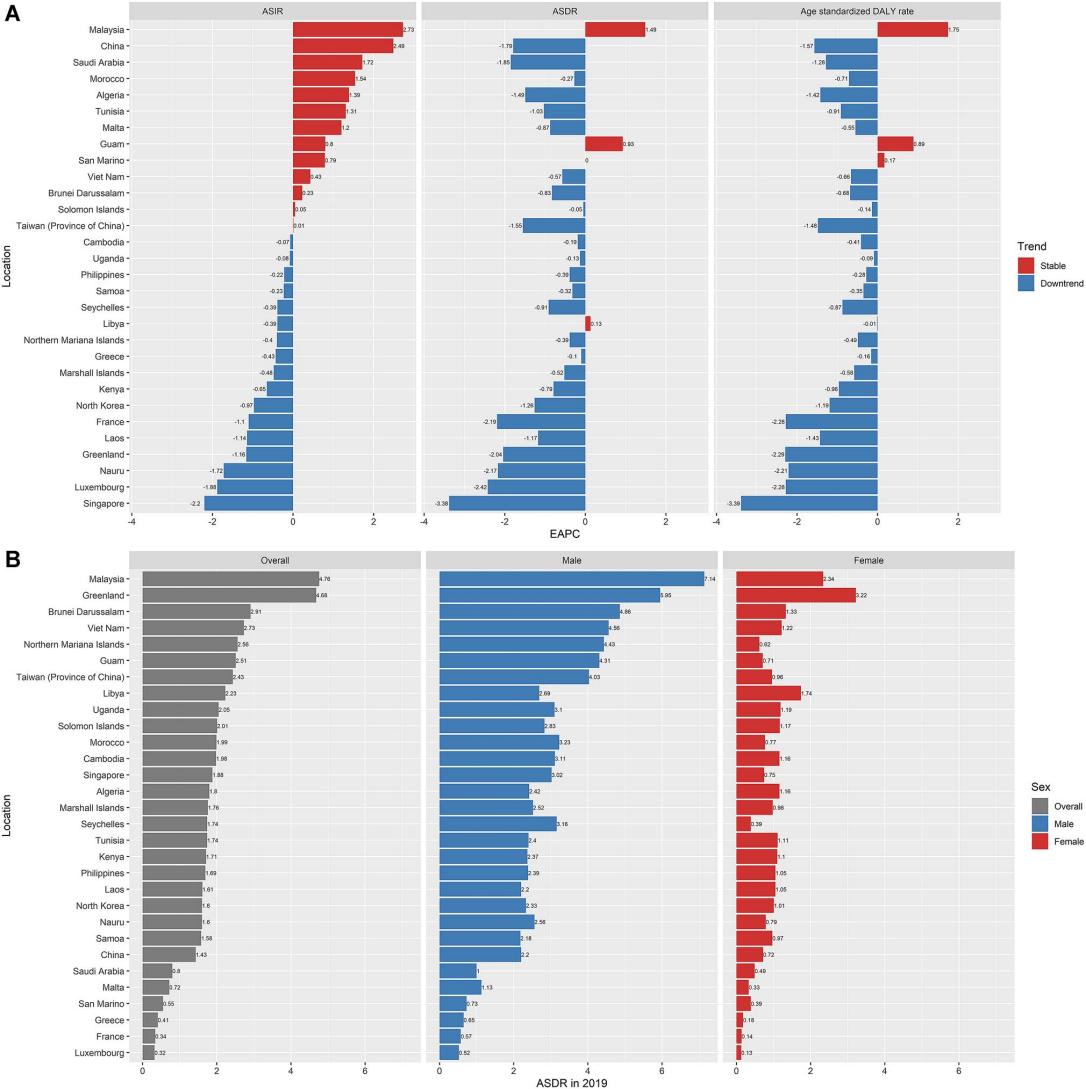
NPC burden of high-incidence territories. **A** EAPCs of ASRs in high-incidence territories. **B** Gender-specific ASDR in high-incidence territories

The ASIR is considered an objective indicator to quantify trends in cancer incidence. We selected the top 30 countries or territories with the highest ASIR of NPC in 2009, and defined them as “High-incidence territories of NPC”. The results showed that while EAPCs of ASIRs were positive in 13 countries and territories, the lower limits of 95% CI of EAPCs in all high-incidence territories were negative, which indicated that although EAPC values were positive, ASIRs of these 13 countries or territories tended to be stable. ASIRs of the remaining 17 countries or territories showed a downward trend (Fig. 5A). Similarly, the lower limits of 95% CI of EAPCs of ASDRs in all 30 territories were negative, indicating that ASDR in high-incidence territories tended to be stable or decreased. Age-standardized DALY rate was the same as ASIR and ASDR (Tables S16–18). Malaysia had the highest ASDR in 2019 (ASDR = 4.76), followed by Greenland (ASDR = 4.68). Among male patients, the highest ASDR was in Malaysia (ASDR = 7.14), while among female patients in Greenland (ASDR = 3.22). The ASDR of male patients was higher than that of female patients (Fig. 5B).

### The relationship between the incidence of NPC and age structure

We divided the onset age of NPC into five age groups: under 10 years, 10–24 years, 25–49 years, 50–74 years and above 75 years, and analyzed the incidence and its rate in different age groups. In general, the number of patients with onset age greater than 50 years accounted for the highest proportion. This phenomenon was most evident in high SDI region (68.71% in 2009 and 71.92% in 2019), and the proportion was yearly increasing. There was no significant difference in age structure between men and women. In addition, we found that the incidence cases of NPC in adolescents could not be ignored in low-middle and low SDI regions (Fig. S2A). Among all age groups, the highest incidence rate was found in patients over 50 years old, especially in the middle-SDI area, while the gender difference was not significant (Fig. S2B).

### Dynamic distribution of risk factors associated with NPC-related mortality

There are many main risk factors associated with NPC, and the GBD database shows three of them: smoking, occupational exposure to carcinogens (mainly formaldehyde), and alcohol use. We found that globally NPC-related death was most closely related to alcohol use from 2009 to 2019, especially in regions with relatively low SDI. The effect of exposure to carcinogens such as formaldehyde was relatively small in all regions and even weaker in regions with relatively high SDI (Figs. S3, S4).

## Discussion

GBD 2019 updates and expands beyond GBD 2017 in ten ways, including update new data from 2018 to 2019, expand the number of countries, add new causes to the modeling framework, revise statistical methods, etc. Compared with the data from 1990 to 2017 in GBD2017, this study uses the data from 2009 to 2019 in GBD2019 to reduce the bias caused by long time span and better reflect the epidemiological trend of NPC in recent years [17]. In this study, we reported the incidence, mortality and DALY data of NPC based on GBD database, and analyzed the prevalence of NPC in the past 11 years by calculating EAPC values. In general, ASIR of NPC increased from 2009 to 2019 worldwide. Only the low SDI region showed a downward trend in ASIR. NPC in high-middle SDI region had the highest incidence cases, while the area with the most deaths was middle-SDI region, which warned about the heavy NPC burden in middle SDI areas. As reported in previous studies, the incidence rate of NPC had been declining in most areas of the world, especially in high-incidence territories such as Asia, Hong Kong and Taiwan [18–20]. The reason for this discrepancy might be that part of researches was relatively old and could not represent the general trend of the last decade. Data from GBD database are more effective, robust and comprehensive, which meets the requirement of accurately describing the epidemic trend of NPC. Another possible reason might be that advances in medical technology led to an increasing number of NPC patients being early detected. Compared with the old screening instruments, the sensitivity and specificity of EBV DNA in plasma samples in screening for NPC were 97.1% and 98.6%, respectively [21]. Careful analysis was necessary while examining global trends in NPC incidence and comparing the results of this study with previous findings. More in-depth global statistics might be needed to verify the global burden of NPC further. In an era when the global NPC situation is still severe, the government and policymakers should raise the awareness of prevention and allocate medical resources rationally to reduce the incidence.

Compared with the increasing incidence rate, the decline in mortality of NPC in the world especially in high-incidence territories was encouraging. We believed this benefited from the advancement in medical professionalism, including enhanced understanding of the pathogenesis and risk factors, precise disease staging systems, individualized chemoradiotherapy (CRT) strategies, and the development of targeted therapy and immunotherapy. There was a significant negative correlation between mortality and SDI, indicating better prognosis of NPC in developed areas, possibly due to more advanced medical means. NPC was highly invasive and metastatic, but sensitive to both chemotherapy and radiotherapy (RT). RT is the mainstay treatment for non-metastatic NPC [1]. In recent years, RT technology has evolved from traditional two-dimensional RT to three-dimensional conformal radiotherapy (3D-CRT), followed by more advanced intensity-modulated radiotherapy (IMRT) and stereotactic body radiotherapy (SBRT). More than 90% of NPC patients achieved excellent local control after receiving high-quality IMRT [22]. For newly diagnosed non-metastatic NPC patients, IMRT reduced the 5-year occurrence rate of locoregional failure to 7.4% [23]. For locally advanced NPC, RT combined with chemotherapy (chemoradiotherapy, CRT) can achieve a better control rate than RT alone, allowing patients to harvest prolonged progression-free survival and overall survival [24]. For recurrent or distant metastatic NPC, immunotherapy has gradually emerged as a promising treatment option that may elevate the treatment of NPC to a new level [25]. Other factors affecting mortality might be changes in lifestyle and reduced exposure to risk factors. The composition of NPC-related risk factors has undergone a series of changes along with the variations of the global environment in the last decade, which may have affected the epidemiological characteristics of NPC to a certain extent.

NPC-related risk factors include genetic factors, EBV infection, and environmental factors such as smoking, drinking, eating salted fish and exposure to carcinogens. EBV latently infects more than 90% of the world population and the majority of NPC are found to be positive for EBV infection [26]. EBV infection may be the most common causal agent of NPC besides genetic factors [1]. Unfortunately, the exact role of EBV in the development of NPC remains unclear [8]. The reason why EBV infection is ubiquitous but NPC is only prevalent in specific regions is still a mystery. Furthermore, considering that the genetic susceptibility to NPC is relatively stable in the population, it is reasonable to assume that the variation trend of NPC was mainly attributed to changes in environmental risk factors [18]. GBD database demonstrates that tobacco and alcohol use are two vital NPC-related risk factors. According to the World Health Organization (WHO), tobacco use in Asian countries such as China, Malaysia, India and Vietnam declined between 2000 and 2020. However, our data showed that ASIR values in these four countries increased gradually from 2009 to 2019. One possible reason is that the positive effects of tobacco control have been obscured by the growth of other risk factors. Further study found that alcohol consumption in many countries such as China, India and Vietnam increased between 2005 and 2016, which might offset the positive effects of smoking control on NPC prevention. What is puzzling is that ASR values of Malaysia rose continuously while its smoking rate was decreasing and its alcohol consumption has not increased in the past 10 years. Further investigations are needed to explain this phenomenon.

By analyzing the data, we found that the NPC mortality was significantly higher among men than women, indicating that female patients had a better prognosis, which was presumably associated with the disease stage at diagnosis, biological behaviors, sex hormones and their receptors [27–29]. A study showed that only 50.6% of female patients had locoregionally advanced NPC, much lower than the 71.9% of male patients [29]. Therefore, female patients might have an earlier disease onset, thereby improving the prognosis to some extent. According to previous studies, the number of male smokers and alcoholics was much higher than female [30, 31]. However, life behaviors and diagnostic delays did not fully explain the gender-specific differences in survival, and the intrinsic biological characteristics of the human body may play a more critical role. Maasberg et al. found in cloning experiments that androgens could triple the proliferation of tumor cells, while estrogens did not promote tumor cell growth [32]. Another study suggested that female sex was a significant protective prognostic factor for NPC regardless of tumor stage. However, this advantage persisted at premenopausal age, declined during menopause and disappeared at postmenopausal period [28]. Other possible explanations for sex-specific differences in prognosis include discrepancies in response to treatment and immune homeostasis [33, 34]. Further studies are required to verify the genuine nature of these potential factors.

The incidence of NPC increase as population ages. It is undisputed that host genetics play an essential role, but the accumulation of mutations with age alone is inadequate to explain why the NPC incidence increases with age. One possible reason is that EBV infection often occurs at an early age, and the long-term stimulation of ubiquitous environmental factors interacts with genetic components, leading to impaired immune control of EBV over the years, thus developing into NPC many years later [5]. A significant proportion of adolescent NPC patients were discovered in the underdeveloped regions, leading to bimodality of the age-incidence curve at those areas, in which the earlier peak (under 25 years) might be associated with HLA gene or other NPC-related susceptibility genes [35].

This is the first study to systematically summarize the global epidemiological trends of NPC in the last decade. However, several deficiencies exist in this study. First, the reliability of the analysis depends on the accuracy and normalization of the data in GBD database. Unavoidably, there may be some undetected potential bias factors in GBD database when dealing with heterogeneous information from different databases. In some underdeveloped areas, obtained data often fail to fully reflect the authenticity of NPC burden because of the immaturity of the disease surveillance and death reporting system. Second, this study lacks sufficient data to determine trends of the disease by histological subtypes. Previous studies found that in the areas where NPC was prevalent, undifferentiated type accounted for the most and was always related to EBV infection, while the differentiated subtypes were more common in non-epidemic areas. Finally, EAPCs are used to evaluate the overall trends on a linear scale and cannot fully reflect the detailed trends of ASRs. To further reduce the global NPC burden, governments and health systems should vigorously advocate the primary prevention of the disease, including control the use of tobacco and alcohol, reduce the consumption of pickled food, increase the intake of fruits and vegetables and take protective measures when exposed to benzene and formaldehyde. In addition, other measures such as large-scale screening, efficient screening strategies, and advanced treatment modalities are also of paramount significance.

## Conclusions

While the increase of nasopharyngeal carcinoma incidence was slight but progressive, mortality of NPC reduced considerably, possibly due to environmental and lifestyle modifications, intensive understanding of risk factors, population screening, precise disease staging systems, and advancement in medical criterion. Subgroup analysis by SDI demonstrated that high-middle and middle SDI regions had the highest burden of NPC. Morbidity and mortality of males significantly outnumbered that of females. The highest incidence occurs in people over 50 years of age, while in underdeveloped territories especially low-middle and low SDI regions, morbidity among adolescent patients could not be underestimated. The next decade should mainly focus on controllable etiological factors, including reducing tobacco and alcohol consumption, controlling the intake of pickled food, and avoiding contact with benzene and formaldehyde. In conclusion, medical policymakers should adjust their medical strategies according to the actual situation of the disease to further reduce the burden of NPC.

## Supplementary Information

Below is the link to the electronic supplementary material.

Supplementary file1 (PNG 420 KB) **Fig.S1 **The age-standardized rates of NPC in 204 countries or territories. (**a**) The ASIR of 204 countries or territories in 2019. (**b**) The ASDR of 204 countries or territories in 2019. (**c**) The age-standardized DALY rate of 204 countries or territories in 2019.

Supplementary file2 (PNG 492 KB) **Fig.S2** The incidence cases and rates of NPC in different age groups from 2009 to 2019. (**a**) The incidence cases of NPC in different age groups in the globe and SDI-related regions. (**b**) The incidence rates of NPC in different age groups in the globe and SDI-related regions.

Supplementary file3 (PNG 500 KB) **Fig.S3** Potential risk factors contributing to NPC-related deaths and DALYs. (**a**) Potential risk factors contributing to NPC-related deaths from 2009 to 2019 in the globe and SDI-related regions. (**b**) Potential risk factors contributing to NPC-related DALY from 2009 to 2019 in the globe and SDI-related regions.

Supplementary file4 (PNG 163 KB) **Fig. S4 **The contribution ratio of potential risk factors for NPC-related deaths and DALYs. (**a**) The contribution ratio of potential risk factors for NPC-related deaths from 2009 to 2019 in the globe and SDI-related regions. (**b**) The contribution ratio of potential risk factors for NPC-related DALYs from 2009 to 2019 in the globe and SDI-related regions.

Supplementary file5 (DOCX 35 KB)

## Data Availability

The datasets generated during and/or analyzed during the current study are available from the Global Health Data Exchange (GHDx) query tool (http://ghdx.healthdata.org/gbd-results-tool).
