# Supplementary figures and images for: The global burden of nasopharyngeal carcinoma from 2009 to 2019: an observational study based on the Global Burden of Disease Study 2019
Source: Eur Arch Otorhinolaryngol. 2021 Jun 19;279(3):1519–33. doi: 10.1007/s00405-021-06922-2 (PMC8897385; doi:10.1007/s00405-021-06922-2)

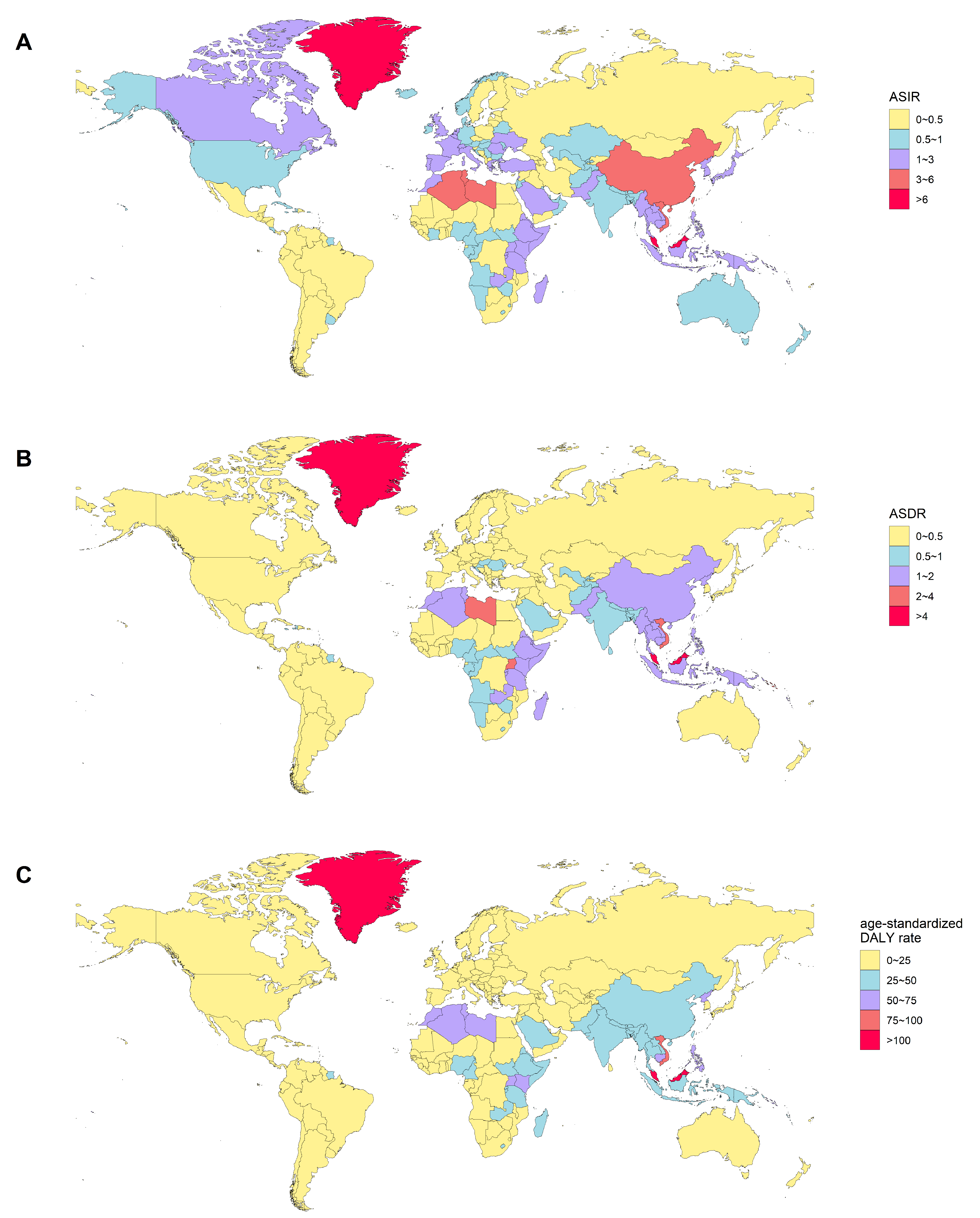

Supplement: Supplementary file 1 — Supplementary file1 (PNG 420 KB) Fig.S1 The age-standardized rates of NPC in 204 countries or territories. (a) The ASIR of 204 countries or territories in 2019. (b) The ASDR of 204 countries or territories in 2019. (c) The age-standardized DALY rate of 204 countries or territories in 2019. [file 405_2021_6922_MOESM1_ESM.png]

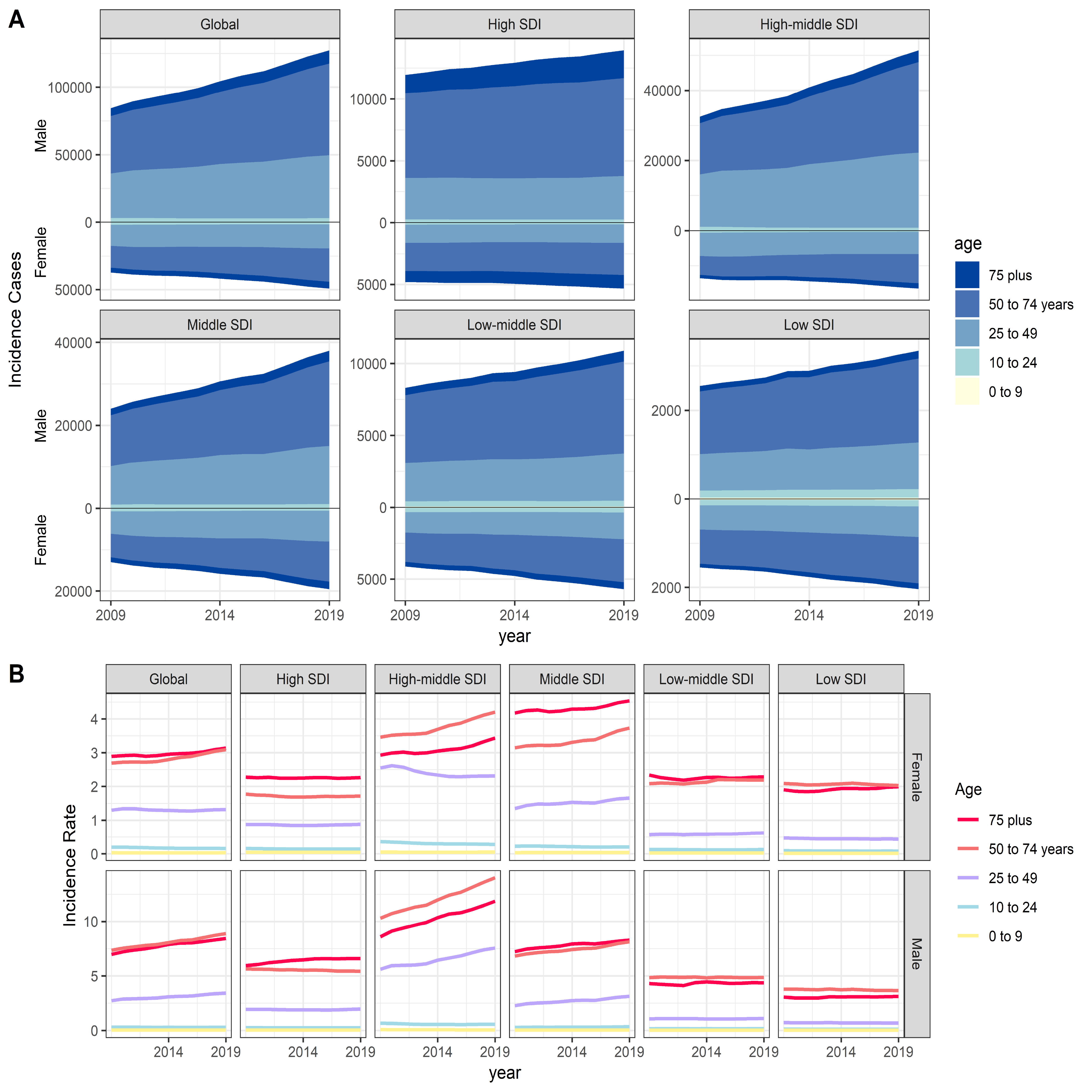

Supplement: Supplementary file 2 — Supplementary file2 (PNG 492 KB) Fig.S2 The incidence cases and rates of NPC in different age groups from 2009 to 2019. (a) The incidence cases of NPC in different age groups in the globe and SDI-related regions. (b) The incidence rates of NPC in different age groups in the globe and SDI-related regions. [file 405_2021_6922_MOESM2_ESM.png]

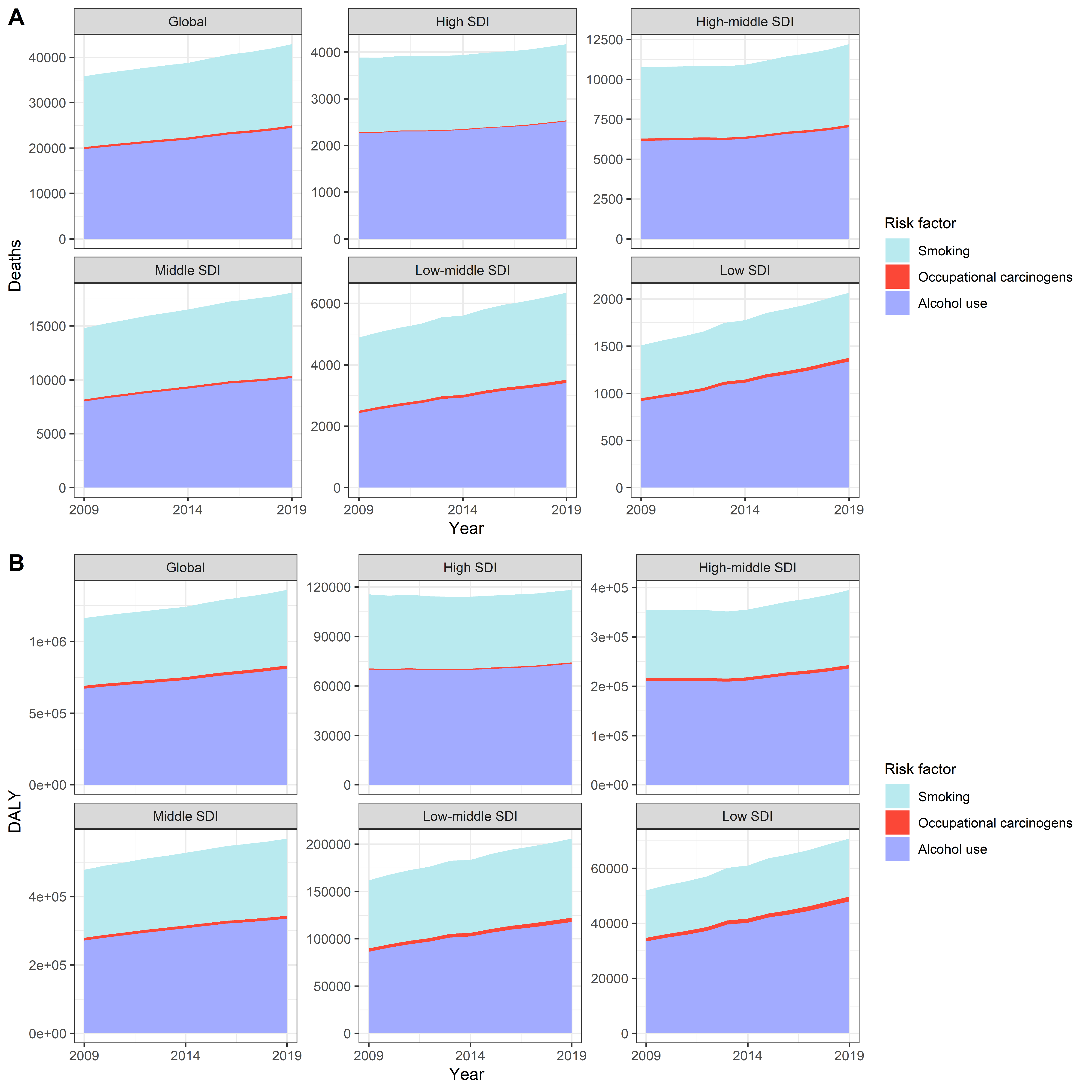

Supplement: Supplementary file 3 — Supplementary file3 (PNG 500 KB) Fig.S3 Potential risk factors contributing to NPC-related deaths and DALYs. (a) Potential risk factors contributing to NPC-related deaths from 2009 to 2019 in the globe and SDI-related regions. (b) Potential risk factors contributing to NPC-related DALY from 2009 to 2019 in the globe and SDI-related regions. [file 405_2021_6922_MOESM3_ESM.png]

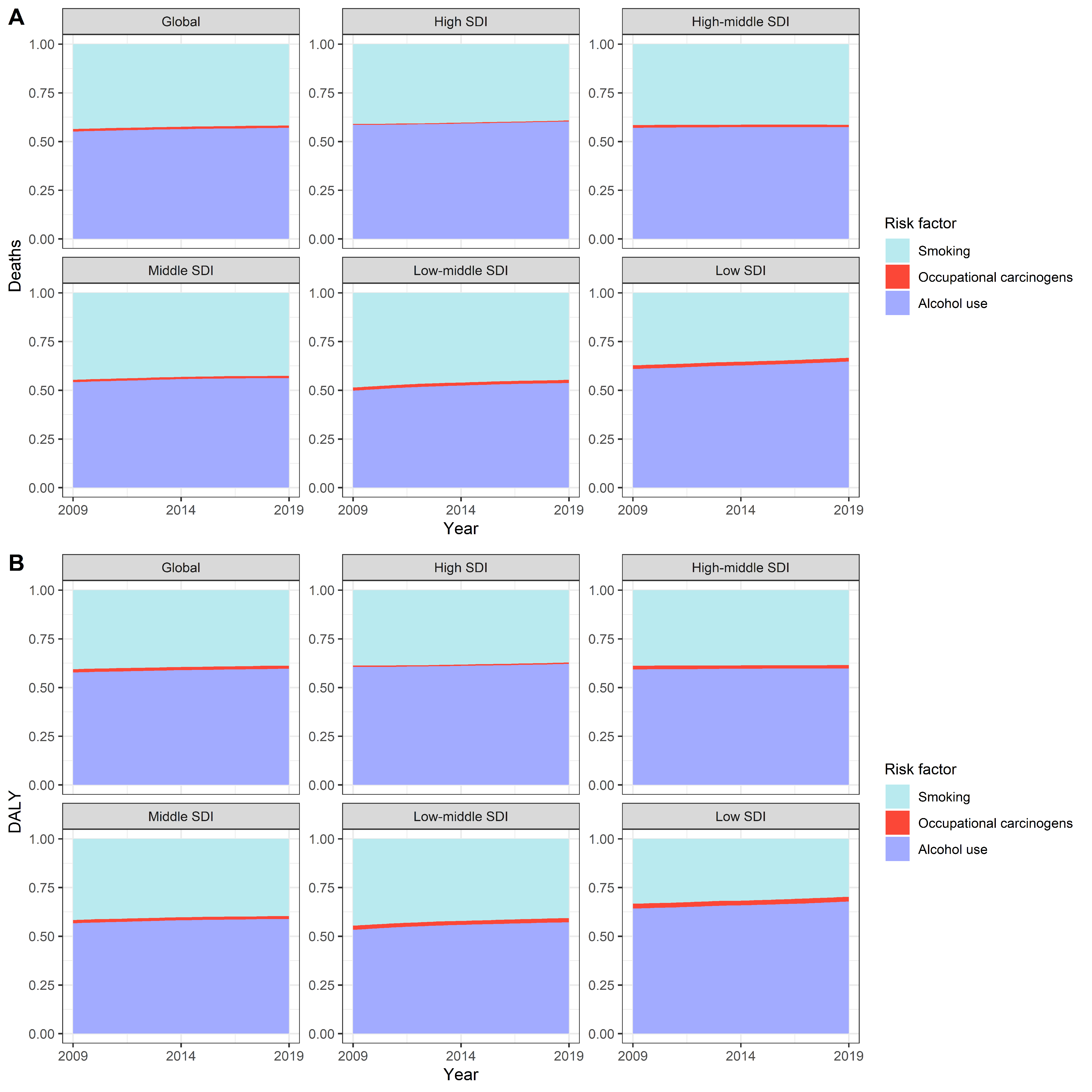

Supplement: Supplementary file 4 — Supplementary file4 (PNG 163 KB) Fig. S4 The contribution ratio of potential risk factors for NPC-related deaths and DALYs. (a) The contribution ratio of potential risk factors for NPC-related deaths from 2009 to 2019 in the globe and SDI-related regions. (b) The contribution ratio of potential risk factors for NPC-related DALYs from 2009 to 2019 in the globe and SDI-related regions. [file 405_2021_6922_MOESM4_ESM.png]
