# Supplementary material for: The global burden of nasopharyngeal carcinoma from 2009 to 2019: an observational study based on the Global Burden of Disease Study 2019
Source: Eur Arch Otorhinolaryngol. 2021 Jun 19;279(3):1519–33. doi: 10.1007/s00405-021-06922-2 (PMC8897385; doi:10.1007/s00405-021-06922-2)
Supplement: Supplementary file 5 — Supplementary file5 (DOCX 35 KB) [file 405_2021_6922_MOESM5_ESM.docx]

**Table S1: Top 20 countries or territories with most incidence cases in 2009.**

| Location | Incidence case |
| --- | --- |
| China | 68784.78 |
| India | 9131.528 |
| Japan | 3737.188 |
| Indonesia | 2796.22 |
| United States of America | 2563.927 |
| Viet Nam | 2440.284 |
| Taiwan (Province of China) | 2030.303 |
| France | 1570.741 |
| Pakistan | 1568.98 |
| Italy | 1364.041 |
| Thailand | 1186.484 |
| Philippines | 1174.602 |
| Germany | 1140.813 |
| Malaysia | 1093.925 |
| Algeria | 1048.802 |
| Spain | 1046.556 |
| Bangladesh | 1044.568 |
| United Kingdom | 1005.195 |
| Turkey | 843.4268 |
| Republic of Korea | 801.3591 |

**Table S2: Top 20 countries or territories with most death cases in 2009.**

| Location | Deaths |
| --- | --- |
| China | 26028.45 |
| India | 8597.774 |
| Indonesia | 2687.397 |
| Viet Nam | 2152.121 |
| Pakistan | 1468.52 |
| Philippines | 1094.727 |
| Bangladesh | 981.1523 |
| United States of America | 968.6423 |
| Thailand | 950.7788 |
| Malaysia | 906.7397 |
| Japan | 862.816 |
| Taiwan (Province of China) | 828.64 |
| Nigeria | 663.1217 |
| Myanmar | 642.4787 |
| Russian Federation | 543.8991 |
| Algeria | 540.7171 |
| Morocco | 530.4939 |
| Democratic People's Republic of Korea | 482.7956 |
| Brazil | 476.6692 |
| France | 414.9958 |

**Table S3: Top 20 countries or territories with highest DALY in 2009.**

| Location | DALY |
| --- | --- |
| China | 858845.8 |
| India | 306535.5 |
| Indonesia | 88623.71 |
| Viet Nam | 74393.23 |
| Pakistan | 56551.26 |
| Philippines | 37629.18 |
| Bangladesh | 35597.29 |
| Malaysia | 31558.8 |
| Thailand | 31197.11 |
| United States of America | 29459.32 |
| Taiwan (Province of China) | 27686.41 |
| Nigeria | 23807.28 |
| Myanmar | 21470.1 |
| Japan | 21008.63 |
| Algeria | 19617.11 |
| Morocco | 18786.09 |
| Russian Federation | 17623.76 |
| Brazil | 17286.73 |
| Democratic People's Republic of Korea | 15945.22 |
| Ethiopia | 14901.79 |

**Table S4: Top 20 countries or territories with highest ASIR in 2009.**

| Location | ASIR |
| --- | --- |
| Singapore | 12.64062 |
| Taiwan (Province of China) | 6.766097 |
| Greenland | 6.739599 |
| Brunei Darussalam | 4.978946 |
| Libya | 4.850366 |
| Malaysia | 4.777524 |
| Tunisia | 4.328989 |
| China | 4.21006 |
| Algeria | 3.574343 |
| Northern Mariana Islands | 3.318551 |
| Viet Nam | 3.134687 |
| Malta | 2.743556 |
| Guam | 2.59749 |
| San Marino | 2.457885 |
| Morocco | 2.391203 |
| Uganda | 2.152799 |
| Seychelles | 2.085748 |
| Solomon Islands | 2.073532 |
| Nauru | 2.025635 |
| Cambodia | 1.968741 |

**Table S5: Top 20 countries or territories with highest ASDR in 2009.**

| Location | ASDR |
| --- | --- |
| Greenland | 5.694761 |
| Malaysia | 4.212119 |
| Brunei Darussalam | 3.097111 |
| Viet Nam | 2.869105 |
| Taiwan (Province of China) | 2.762961 |
| Northern Mariana Islands | 2.711405 |
| Singapore | 2.471426 |
| Libya | 2.261185 |
| Guam | 2.241325 |
| Uganda | 2.113943 |
| Algeria | 2.087369 |
| Solomon Islands | 2.024893 |
| Morocco | 2.009578 |
| Cambodia | 1.984631 |
| Nauru | 1.981836 |
| Tunisia | 1.924807 |
| Seychelles | 1.921639 |
| Marshall Islands | 1.854407 |
| Kenya | 1.826235 |
| Lao People's Democratic Republic | 1.798169 |

**Table S6: Top 20 countries or territories with highest age-standardized DALY rate in 2009.**

| Location | Age standardized DALY |
| --- | --- |
| Greenland | 171.9936 |
| Malaysia | 131.3115 |
| Brunei Darussalam | 92.92667 |
| Viet Nam | 91.58707 |
| Taiwan (Province of China) | 91.23103 |
| Singapore | 81.71189 |
| Northern Mariana Islands | 79.82748 |
| Libya | 72.84339 |
| Guam | 69.08708 |
| Uganda | 68.59974 |
| Algeria | 67.07621 |
| Solomon Islands | 66.17058 |
| Morocco | 65.55759 |
| Nauru | 61.55867 |
| Tunisia | 60.72423 |
| Kenya | 59.7122 |
| Cambodia | 58.43494 |
| Marshall Islands | 57.78185 |
| Seychelles | 57.71578 |
| Democratic People's Republic of Korea | 56.86443 |

**Table S7: Top 20 countries or territories with most incidence cases in 2019.**

| Location | Incidence case |
| --- | --- |
| China | 110432.9 |
| India | 12212.34 |
| Japan | 3984.616 |
| Viet Nam | 3460.404 |
| Indonesia | 3356.389 |
| United States of America | 2869.841 |
| Taiwan (Province of China) | 2379.615 |
| Pakistan | 2012.067 |
| Malaysia | 1845.589 |
| Algeria | 1687.496 |
| France | 1578.018 |
| Philippines | 1558.8 |
| Thailand | 1539.225 |
| Italy | 1423.896 |
| Bangladesh | 1304.271 |
| Germany | 1210.85 |
| Spain | 1170.384 |
| United Kingdom | 1121.257 |
| Republic of Korea | 1086.86 |
| Turkey | 1068.887 |

**Table S8: Top 20 countries or territories with most death cases in 2019.**

| Location | Deaths |
| --- | --- |
| China | 28659.47 |
| India | 11358.14 |
| Indonesia | 3216.984 |
| Viet Nam | 2788.086 |
| Pakistan | 1858.626 |
| Philippines | 1431.901 |
| Malaysia | 1379.532 |
| Bangladesh | 1210.275 |
| Thailand | 1175.702 |
| United States of America | 1126.503 |
| Japan | 935.7032 |
| Taiwan (Province of China) | 918.6049 |
| Nigeria | 884.9876 |
| Myanmar | 692.7666 |
| Morocco | 674.809 |
| Algeria | 658.5829 |
| Russian Federation | 535.0108 |
| Brazil | 527.0485 |
| Democratic People's Republic of Korea | 525.3037 |
| Ethiopia | 507.3653 |

**Table S9: Top 20 countries or territories with highest DALY in 2019.**

| Location | DALY |
| --- | --- |
| China | 912106.5 |
| India | 388094.1 |
| Indonesia | 100796.6 |
| Viet Nam | 93450.42 |
| Pakistan | 71646.92 |
| Philippines | 48257.53 |
| Malaysia | 46906.14 |
| Bangladesh | 40919.67 |
| Thailand | 35202.7 |
| United States of America | 32364.16 |
| Nigeria | 32220.6 |
| Taiwan (Province of China) | 29183.42 |
| Algeria | 23275.83 |
| Morocco | 22370.16 |
| Myanmar | 22100.57 |
| Japan | 20308.03 |
| Ethiopia | 18446.98 |
| Brazil | 18249.43 |
| Kenya | 17393.54 |
| Democratic People's Republic of Korea | 17040.88 |

**Table S10: Top 20 countries or territories with highest ASIR in 2019.**

| Location | ASIR |
| --- | --- |
| Singapore | 10.81277 |
| Taiwan (Province of China) | 7.139678 |
| Malaysia | 6.082743 |
| Greenland | 6.000956 |
| China | 5.653457 |
| Brunei Darussalam | 5.303124 |
| Tunisia | 4.940545 |
| Libya | 4.753241 |
| Algeria | 4.176737 |
| Viet Nam | 3.28075 |
| Northern Mariana Islands | 3.122871 |
| Malta | 3.016266 |
| Guam | 2.899198 |
| Morocco | 2.704011 |
| San Marino | 2.650744 |
| Uganda | 2.100102 |
| Solomon Islands | 2.081452 |
| Saudi Arabia | 2.077754 |
| Seychelles | 2.001319 |
| Cambodia | 1.986586 |

**Table S11: Top 20 countries or territories with highest ASDR in 2019.**

| Location | ASDR |
| --- | --- |
| Malaysia | 4.762839 |
| Greenland | 4.676259 |
| Brunei Darussalam | 2.909538 |
| Viet Nam | 2.726088 |
| Northern Mariana Islands | 2.556061 |
| Guam | 2.505056 |
| Taiwan (Province of China) | 2.434649 |
| Libya | 2.226967 |
| Uganda | 2.054057 |
| Solomon Islands | 2.014647 |
| Morocco | 1.991178 |
| Cambodia | 1.97662 |
| Singapore | 1.880012 |
| Algeria | 1.799844 |
| Marshall Islands | 1.763827 |
| Tunisia | 1.741786 |
| Seychelles | 1.737289 |
| Kenya | 1.710697 |
| Philippines | 1.690374 |
| Lao People's Democratic Republic | 1.607308 |

**Table S12: Top 20 countries or territories with highest age-standardized DALY rate in 2019.**

| Location | Age standardized DALY |
| --- | --- |
| Malaysia | 152.2872 |
| Greenland | 137.7435 |
| Brunei Darussalam | 89.84702 |
| Viet Nam | 86.355 |
| Taiwan (Province of China) | 81.4516 |
| Guam | 77.55027 |
| Northern Mariana Islands | 74.3652 |
| Libya | 70.90261 |
| Uganda | 66.72233 |
| Solomon Islands | 65.33631 |
| Singapore | 61.83486 |
| Morocco | 61.82838 |
| Algeria | 58.04645 |
| Cambodia | 57.16202 |
| Tunisia | 55.58397 |
| Kenya | 55.03619 |
| Marshall Islands | 54.66335 |
| Seychelles | 52.9685 |
| Samoa | 52.44351 |
| Democratic People's Republic of Korea | 51.40326 |

**Table S13: Top 20 countries or territories with the highest EAPC of ASIR.**

| Location | EAPC-ASIR | Upper limits | Low limits |
| --- | --- | --- | --- |
| Bulgaria | 3.87255643 | 13.6110457 | -5.0311709 |
| Ukraine | 3.6756132 | 4.87354792 | 2.49136208 |
| Costa Rica | 2.95071453 | 8.29094081 | -2.1261655 |
| Malaysia | 2.72881975 | 7.91733346 | -2.2102375 |
| China | 2.48671838 | 7.58535992 | -2.3702904 |
| Kyrgyzstan | 2.07446645 | 7.40942937 | -2.995512 |
| Palestine | 1.94059955 | 2.83065516 | 1.05824785 |
| Burkina Faso | 1.92949732 | 8.72023443 | -4.4370859 |
| Chile | 1.75148501 | 5.52759002 | -1.8894992 |
| Saudi Arabia | 1.72450202 | 4.8786369 | -1.334775 |
| Kazakhstan | 1.67443624 | 6.71481512 | -3.1278743 |
| Lebanon | 1.65122909 | 2.08589099 | 1.2184179 |
| Cuba | 1.61769382 | 7.81768157 | -4.2257675 |
| Nicaragua | 1.59101408 | 7.23005434 | -3.751479 |
| Morocco | 1.53533069 | 5.10541625 | -1.9134908 |
| Belgium | 1.51863816 | 5.5264185 | -2.336931 |
| Timor-Leste | 1.48401144 | 3.92958675 | -0.9040168 |
| Algeria | 1.39497009 | 3.41894438 | -0.5893937 |
| Cabo Verde | 1.36640843 | 13.0655419 | -9.1221907 |
| Bulgaria | 3.87255643 | 13.6110457 | -5.0311709 |

**Table S14: Top 20 countries or territories with the highest EAPC of ASDR.**

| Location | EAPC-ASDR | Upper limits | Low limits |
| --- | --- | --- | --- |
| Ukraine | 2.1263448 | 10.9395827 | -5.9867538 |
| Bulgaria | 2.04777652 | 8.8974656 | -4.3710647 |
| Burkina Faso | 1.83185821 | 8.53746686 | -4.4594678 |
| Kyrgyzstan | 1.77709637 | 7.0222764 | -3.2110165 |
| Malaysia | 1.48571984 | 6.91842675 | -3.670942 |
| Timor-Leste | 1.33902107 | 3.78879393 | -1.0529287 |
| Saint Lucia | 1.17293946 | 6.45374135 | -3.8459001 |
| Nepal | 1.03270378 | 3.0496744 | -0.9447891 |
| Costa Rica | 0.9966806 | 7.45293901 | -5.0716566 |
| Gambia | 0.99484968 | 5.57805354 | -3.3893947 |
| Uzbekistan | 0.95303145 | 4.34986124 | -2.3332237 |
| Guam | 0.92558508 | 10.0026515 | -7.4024709 |
| Cabo Verde | 0.86492877 | 12.6133948 | -9.6578709 |
| Saint Vincent and the Grenadines | 0.82971 | 3.11507154 | -1.4050006 |
| Kazakhstan | 0.8153982 | 5.97652537 | -4.0943787 |
| Comoros | 0.66290725 | 3.04184801 | -1.6611106 |
| Senegal | 0.6198259 | 6.79883861 | -5.2016904 |
| Fiji | 0.59333452 | 3.02001895 | -1.7761882 |
| Cuba | 0.49410583 | 7.57644944 | -6.1219685 |
| Nicaragua | 0.44755171 | 6.77477497 | -5.5047351 |

**Table S15: Top 20 countries or territories with the highest EAPC of age-standardized DALY rate.**

| Location | EAPC-DALY rate | Upper limits | Low limits |
| --- | --- | --- | --- |
| Ukraine | 3.3397228 | 11.2647193 | -4.020804 |
| Bulgaria | 2.04752231 | 9.45910163 | -4.862212 |
| Burkina Faso | 2.04386939 | 9.01119792 | -4.4781502 |
| Malaysia | 1.74874879 | 7.25021751 | -3.4705186 |
| Timor-Leste | 1.52651459 | 4.07183544 | -0.9565545 |
| Kyrgyzstan | 1.467786 | 6.79305019 | -3.5919325 |
| Costa Rica | 1.3760078 | 7.58934444 | -4.4785056 |
| Comoros | 1.16817785 | 3.45162107 | -1.0648639 |
| Gambia | 1.01853202 | 5.61100566 | -3.3742388 |
| Uzbekistan | 0.97429886 | 4.2949794 | -2.2406535 |
| Guam | 0.88910105 | 9.78468382 | -7.2856945 |
| Nepal | 0.7545441 | 2.47843643 | -0.9403489 |
| Saint Lucia | 0.75180674 | 6.02039004 | -4.2549593 |
| Senegal | 0.70806609 | 7.22067658 | -5.4089668 |
| Saint Vincent and the Grenadines | 0.70760682 | 3.08706169 | -1.6169255 |
| Niger | 0.66585534 | 9.35689626 | -7.3344729 |
| Nicaragua | 0.58776561 | 6.23185687 | -4.7564555 |
| Fiji | 0.52192119 | 2.72408753 | -1.6330358 |
| Cuba | 0.48397984 | 7.72322867 | -6.2687748 |
| Suriname | 0.37048325 | 3.49876846 | -2.6632485 |

**Table S16: EAPC of ASIR and its 95% CI in high incidence territories.**

| Location | EAPC-ASIR | Upper limits | Low limits |
| --- | --- | --- | --- |
| Singapore | -2.2013 | 4.222958 | -8.22957 |
| Taiwan (Province of China) | 0.006359 | 5.889689 | -5.55009 |
| Greenland | -1.15877 | 0.996432 | -3.26799 |
| Brunei Darussalam | 0.234067 | 5.977013 | -5.19767 |
| Libya | -0.38545 | 3.17919 | -3.82694 |
| Malaysia | 2.72882 | 7.917333 | -2.21024 |
| Tunisia | 1.314706 | 4.052295 | -1.35086 |
| China | 2.486718 | 7.58536 | -2.37029 |
| Algeria | 1.39497 | 3.418944 | -0.58939 |
| Northern Mariana Islands | -0.39998 | 8.769816 | -8.79672 |
| Viet Nam | 0.433776 | 6.593794 | -5.37026 |
| Malta | 1.203577 | 5.633594 | -3.04066 |
| Guam | 0.799355 | 9.352871 | -7.08511 |
| San Marino | 0.791295 | 2.848409 | -1.22467 |
| Morocco | 1.535331 | 5.105416 | -1.91349 |
| Uganda | -0.08446 | 4.681209 | -4.63316 |
| Seychelles | -0.38545 | 10.07899 | -9.8551 |
| Solomon Islands | 0.046114 | 4.240309 | -3.97932 |
| Nauru | -1.71748 | 3.668469 | -6.82361 |
| Cambodia | -0.06829 | 4.430626 | -4.37339 |
| North Korea | -0.97023 | 2.771467 | -4.5757 |
| Marshall Islands | -0.47998 | 3.956871 | -4.72747 |
| Greece | -0.42941 | 4.520543 | -5.14493 |
| Philippines | -0.22087 | 3.717237 | -4.00945 |
| Laos | -1.14191 | 2.188369 | -4.36366 |
| Luxembourg | -1.87635 | 3.623331 | -7.08414 |
| Kenya | -0.64561 | 3.45812 | -4.58657 |
| France | -1.10234 | 3.997988 | -5.95253 |
| Samoa | -0.22549 | 3.544012 | -3.85776 |
| Saudi Arabia | 1.724502 | 4.878637 | -1.33477 |

**Table S17: EAPC of ASDR and its 95% CI in high incidence territories.**

| Location | EAPC-ASDR | Upper limits | Low limits |
| --- | --- | --- | --- |
| Singapore | -3.37864 | 3.361269 | -9.67906 |
| Taiwan (Province of China) | -1.55243 | 5.243838 | -7.90983 |
| Greenland | -2.0393 | 0.859655 | -4.85493 |
| Brunei Darussalam | -0.83306 | 5.24016 | -6.55581 |
| Libya | 0.12762 | 2.08677 | -1.79393 |
| Malaysia | 1.48572 | 6.918427 | -3.67094 |
| Tunisia | -1.02959 | 2.596051 | -4.52711 |
| China | -1.7923 | 3.453887 | -6.77245 |
| Algeria | -1.48626 | 1.990893 | -4.84487 |
| Northern Mariana Islands | -0.38535 | 9.556127 | -9.4247 |
| Viet Nam | -0.57329 | 5.76002 | -6.52734 |
| Malta | -0.87004 | 5.091886 | -6.49375 |
| Guam | 0.925585 | 10.00265 | -7.40247 |
| San Marino | 0.000465 | 2.849944 | -2.77007 |
| Morocco | -0.27377 | 6.439411 | -6.56355 |
| Uganda | -0.13161 | 4.627255 | -4.67402 |
| Seychelles | -0.90838 | 9.718141 | -10.5057 |
| Solomon Islands | -0.05111 | 4.21072 | -4.13864 |
| Nauru | -2.17445 | 3.27712 | -7.33825 |
| Cambodia | -0.19315 | 4.353852 | -4.54202 |
| North Korea | -1.26346 | 2.645536 | -5.0236 |
| Marshall Islands | -0.52261 | 3.942429 | -4.79585 |
| Greece | -0.09746 | 6.266898 | -6.08066 |
| Philippines | -0.39124 | 3.500589 | -4.13673 |
| Laos | -1.16598 | 2.190209 | -4.41195 |
| Luxembourg | -2.42335 | 4.222673 | -8.64558 |
| Kenya | -0.79227 | 2.985639 | -4.43159 |
| France | -2.18864 | 4.400943 | -8.3623 |
| Samoa | -0.31754 | 3.600539 | -4.08744 |
| Saudi Arabia | -1.85217 | 1.568276 | -5.15743 |

**Table S18: EAPC of age-standardized DALY rate and its 95% CI in high incidence territories.**

| Location | EAPC-DALY rate | Upper limits | Low limits |
| --- | --- | --- | --- |
| Singapore | -3.38913 | 3.122479 | -9.48956 |
| Taiwan (Province of China) | -1.4841 | 5.344697 | -7.87024 |
| Greenland | -2.29414 | 0.329243 | -4.84893 |
| Brunei Darussalam | -0.68157 | 5.040696 | -6.09211 |
| Libya | -0.01245 | 1.704412 | -1.70034 |
| Malaysia | 1.748749 | 7.250218 | -3.47052 |
| Tunisia | -0.90642 | 2.225221 | -3.94212 |
| China | -1.57365 | 3.781285 | -6.65228 |
| Algeria | -1.41735 | 1.732298 | -4.46948 |
| Northern Mariana Islands | -0.48577 | 9.291813 | -9.38862 |
| Viet Nam | -0.6605 | 5.743371 | -6.67655 |
| Malta | -0.54875 | 5.30504 | -6.07713 |
| Guam | 0.889101 | 9.784684 | -7.28569 |
| San Marino | 0.168306 | 3.040371 | -2.62371 |
| Morocco | -0.71284 | 5.550368 | -6.6044 |
| Uganda | -0.08736 | 4.733234 | -4.68608 |
| Seychelles | -0.86532 | 9.918725 | -10.5914 |
| Solomon Islands | -0.14037 | 3.891583 | -4.01584 |
| Nauru | -2.21438 | 3.187248 | -7.33324 |
| Cambodia | -0.41386 | 4.097024 | -4.72927 |
| North Korea | -1.18636 | 2.635051 | -4.86548 |
| Marshall Islands | -0.57517 | 3.743819 | -4.71436 |
| Greece | -0.16222 | 6.271782 | -6.2067 |
| Philippines | -0.27818 | 3.904744 | -4.29271 |
| Laos | -1.42709 | 1.879426 | -4.62629 |
| Luxembourg | -2.27925 | 4.350509 | -8.48779 |
| Kenya | -0.96032 | 2.756857 | -4.54304 |
| France | -2.28484 | 4.123318 | -8.29861 |
| Samoa | -0.35297 | 3.433056 | -4.00041 |
| Saudi Arabia | -1.2814 | 1.515008 | -4.00078 |
